# Hexakis(2,3,6-tri-*O*-methyl)-α-cyclodextrin–I_5_^–^ complex in aqueous I^–^/I_3_^–^ thermocells and enhancement in the Seebeck coefficient†
†Electronic supplementary information (ESI) available. See DOI: 10.1039/c8sc03821j


**DOI:** 10.1039/c8sc03821j

**Published:** 2018-10-22

**Authors:** Yimin Liang, Teppei Yamada, Hongyao Zhou, Nobuo Kimizuka

**Affiliations:** a Division of Chemistry and Biochemistry , Graduate School of Engineering , Kyushu University , Motooka 744, Nishi-ku , Fukuoka 819-0395 , Japan . Email: teppei343@gmail.com; b Center for Molecular Systems , Kyushu University , Motooka 744, Nishi-ku , Fukuoka 819-0395 , Japan

## Abstract

A large Seebeck coefficient (*S*_e_) of 1.9 mV K^–1^ was recorded for the I^–^/I_3_^–^ thermocell by utilizing the host-guest complexation of hexakis(2,3,6-tri-*O*-methyl)-α-cyclodextrin (Me_18_-α-CD) with the oxidized iodide species. The thermocell measurement and UV-vis spectroscopy unveiled the formation of an Me_18_-α-CD–pentaiodide (I_5_^–^) complex, which is in remarkable contrast to the triiodide complex α-CD–I_3_^–^ previously reported. Although the precipitation of the α-CD–I_3_^–^ complex in the presence of an electrolyte such as potassium chloride is a problem in thermocells, this issue was solved by using Me_18_-α-CD as a host compound. The absence of precipitation in the Me_18_-α-CD and I^–^/I_3_^–^ system containing potassium chloride not only improved the *S*_e_ of the I^–^/I_3_^–^ thermocell, but also significantly enhanced the temporal stability of its power output. This is the first observation that I_5_^–^ species is formed in aqueous solution in a thermocell. Furthermore, the solution equilibrium of the redox couples was controlled by tuning the chemical structure of the host compounds. Thus, the integration of host-guest chemistry with redox couples extends the application of thermocells.

## Abstract 2

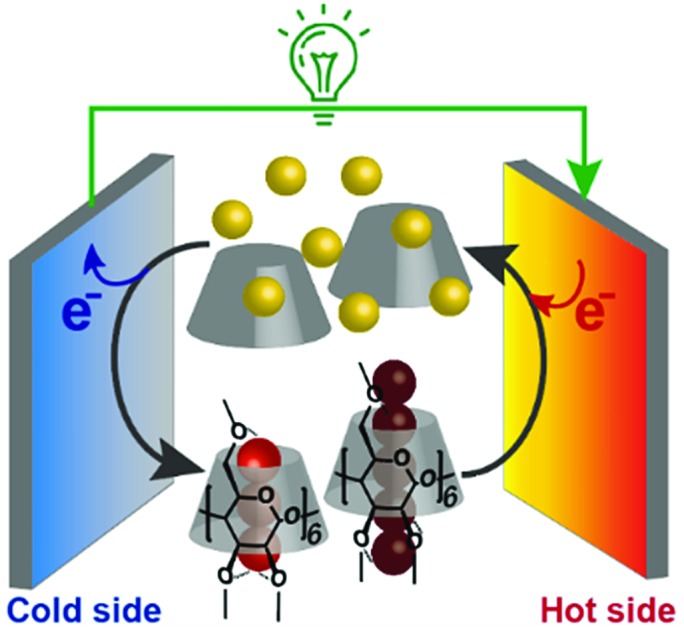
Supramolecular thermocell composed of I^–^ (yellow balls), I_3_^–^ (trio of red balls), I_5_^–^ (five connected dark red balls) and Me_18_-α-CD (gray cone-shaped cylinder).

## Introduction

Thermo-electric conversion based on the Seebeck effect has attracted much interest due to its potential to retrieve waste heat and convert it to electricity, which provides a promising way to reduce the consumption of fossil fuel. Accordingly, semiconductor-based devices have been utilized as the main thermo-electric materials for many years. However, their small *S*_e_ limits their development.^1^–^5^ Thermocells, often referred to as thermo-electrochemical cells or thermo-galvanic cells, offer an alternative approach for the design of thermo-electric devices, which have attracted increasing attention due to their relatively high *S*_e_ and low cost.^6^–^8^

Thermocells are composed of a redox pair that is dissolved into the electrolyte. As reviewed by Quikenden, Pringle *et al.*, various strategies have been devoted to improving the performance of thermocells, and their *S*_e_ reached up to 1.4 mV K^–1^ using Pt electrodes and an aqueous solution of [Fe(CN)_6_]^3–/4–^.^9^–^11^ Recently, carbon nanotubes were utilized as electrodes and the conversion efficiency of the thermocell was enhanced to 3.95% relative to the Carnot cycle.^12^–^14^ Ionic liquid-based thermocells have also been extensively studied, which exhibited a high *S*_e_ of 2.2 mV K^–1^ in a wide temperature range.^15^–^17^ However, strategies to improve the figure-of-merit value is still required for the practical usage of thermocells.

We recently reported the concept of a supramolecular thermocell, which was demonstrated by introducing α-cyclodextrin (α-CD, Fig. S1a†) as a molecular host to the I^–^/I_3_^–^ thermocell. α-CD selectively captured the hydrophobic I_3_^–^ anion, which led to a significant enhancement of *S*_e_ from 0.8 to 1.4 mV K^–1^.^18^ The addition of KCl as the supporting electrolyte resulted in the precipitation of the α-CD–I_3_^–^ complex in the lower-temperature cells, which further increased the *S*_e_ value to *ca.* 2 mV K^–1^. Polymers such as starch and poly(vinylpyrrolidone) also served as host matrices, which resulted in an increase in *S*_e_ to 1.5 and 1.2 mV K^–1^, respectively.^19^ This host–guest approach is applicable to various types of redox species and its effect is independent of the type of electrode. This was useful in improving the *S*_e_ value in thermocells, and the highest *S*_e_ of *ca.* 2 mV K^–1^ was achieved for the precipitation–dissolution equilibrium system of α-CD–I_3_^–^ in aqueous KCl.

Although the addition of KCl was desirable to increase the conductivity of the electrochemical thermocell, the precipitation observed for the α-CD–I_3_^–^ system decreased the diffusion ratio of the redox species and also impaired the durability of the thermocell. Thus, to solve this issue, it is essential to develop host molecules that show enhanced stability with the inclusion complex in aqueous electrolyte. Herein, we report the use of hexakis(2,3,6-tri-*O*-methyl)-α-cyclodextrin (Me_18_-α-CD, Fig. S1c†) as a suitable host molecule for I^–^/I_3_^–^ thermocells. The aqueous solution of Me_18_-α-CD and iodide showed high stability and no precipitation was observed even in the presence of supporting electrolyte. The *S*_e_ of the thermocell reached 1.92 mV K^–1^, which is the highest value reported to date for homogeneous I^–^/I_3_^–^ thermocells.

**Fig. 1:**
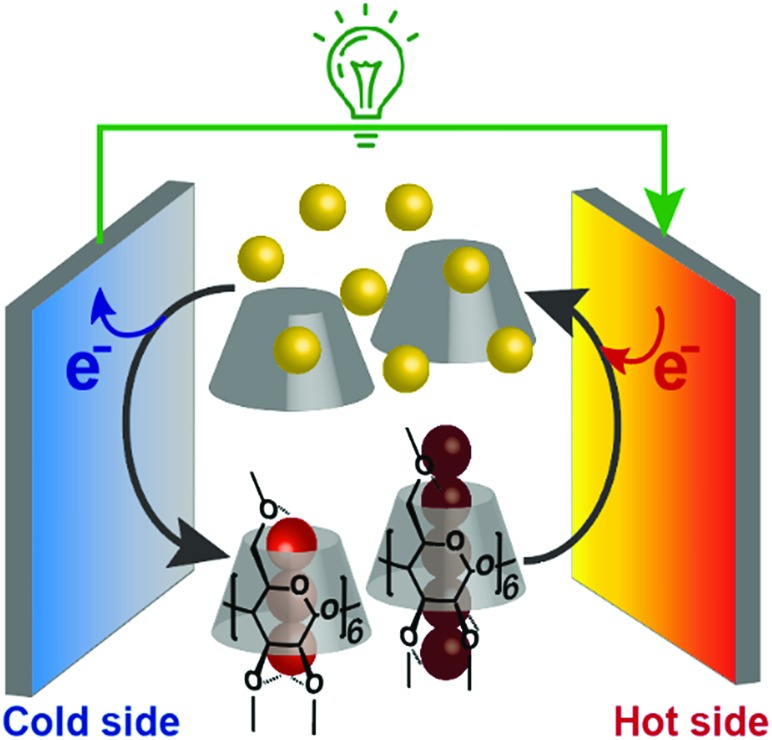
Schematic of a supramolecular thermocell composed of I^–^ (yellow balls), I_3_^–^ (trio of red balls), I_5_^–^ (five connected dark red balls) and Me_18_-α-CD (gray cone-shaped cylinder).

In addition, we found that this system showed an unusual off-stoichiometric interaction between Me_18_-α-CD and I_3_^–^, and the formation of Me_18_-α-CD–pentaiodide (I_5_^–^) complex was demonstrated for the first time. It should be noted that the formation of I_5_^–^ species in aqueous solution has never been confirmed, and its presence has been only reported for solid crystals.^20^–^23^ This result shows the design of proper host molecules leads to superior thermoelectric conversion (Fig. 1).

## Results and discussion

### Thermocell measurement

**Fig. 2:**
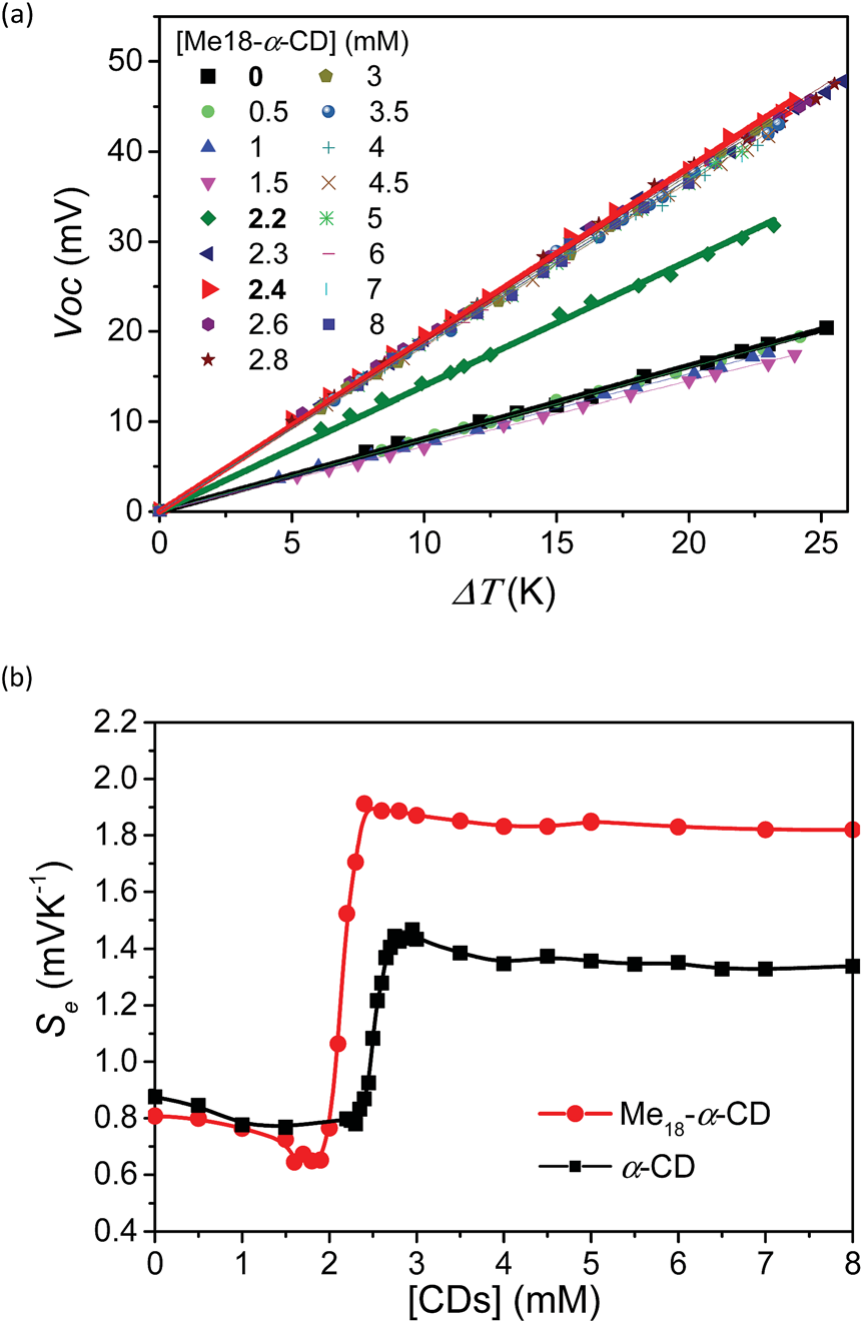
(a) Plots of *V*_oc_ and Δ*T* with various Me_18_-α-CD concentrations. [KI]_0_ = 12.5 mM and [I_2_]_0_ = 2.5 mM. (b) Seebeck coefficient estimated from the slope of (a) with various concentrations of Me_18_-α-CD (red circles) and α-CD (black squares) in the thermocells. The margin of error for each point was less than 0.02 mV K^–1^.

To investigate the effect of host–guest interaction, I^–^/I_3_^–^ thermocells were prepared using various concentrations of Me_18_-α-CD. The concentration of the redox couple was kept same as that in the previous study ([KI] = 10 mM and [I_3_^–^] = 2.5 mM).^18^ In contrast to the previous α-CD–I^–^/I_3_^–^ system, the present Me_18_-α-CD did not cause precipitation, even in the presence of KCl. The detail of the experimental procedure is described in the SI. The open circuit voltage (*V*_oc_) of the cell between the hot and cold electrodes corresponds to the generating voltage of the cell. The temperature dependence of *V*_oc_ at varied concentrations of hosts is shown in Fig. 2a. The *V*_oc_ values were proportional to the temperature difference (Δ*T*), where the slope of the line corresponds to the Seebeck coefficient. That is, a high *S*_e_ value indicates a large voltage with the same temperature difference. In Fig. 2b, the obtained *S*_e_ was plotted as a function of the Me_18_-α-CD and α-CD concentration. The data for the α-CD–I^–^/I_3_^–^ system was also shown for comparison. The *S*_e_ value obtained without hosts was 0.84 mV K^–1^, which confirms the reproducibility of the previous study (0.86 mV K^–1^).^18^ In the case of the Me_18_-α-CD–I^–^/I_3_^–^ system, its *S*_e_ value was almost constant below the Me_18_-α-CD concentration of 1.5 mM, while a drastic increase was observed above the concentration of 2.0 mM. The high *S*_e_ value was maintained above the concentration of *ca.* 2.4 mM. The maximum *S*_e_ of 1.92 mV K^–1^ was observed at the concentration of 2.2 mM, which is 1.08 mV K^–1^ higher than that without host molecules. This value is also *ca.* 0.5 mV K^–1^ higher than that obtained from the pristine α-CD–I^–^/I_3_^–^ cell system in the absence of KCl and is the highest value observed for homogeneous I^–^/I_3_^–^ thermocells. Although we reported a slightly higher *S*_e_ value of 1.97 mV K^–1^ for inhomogeneous precipitate–dissolution equilibrium mixtures caused by the addition of KCl to the α-CD–I^–^/I_3_^–^ thermocell, the presence of these precipitates significantly decreased its durability.^18^

An inflection point was observed for the curve at [Me_18_-α-CD] = *ca.* 2.1 mM, which is well below that observed for α-CD (*ca.* 2.5 mM). In the case of α-CD, it stoichiometrically captures I_3_^–^, thus the concentration of the inflection point was almost the same as the initial concentration of I_3_^–^. The observed shift in the inflection point for Me_18_-α-CD reflects the formation of complexes with different stoichiometries. As discussed below, the observed shift is derived from the complexation of Me_18_-α-CD with I_5_^–^ species.

### Isothermal titration calorimetry

**Fig. 3:**
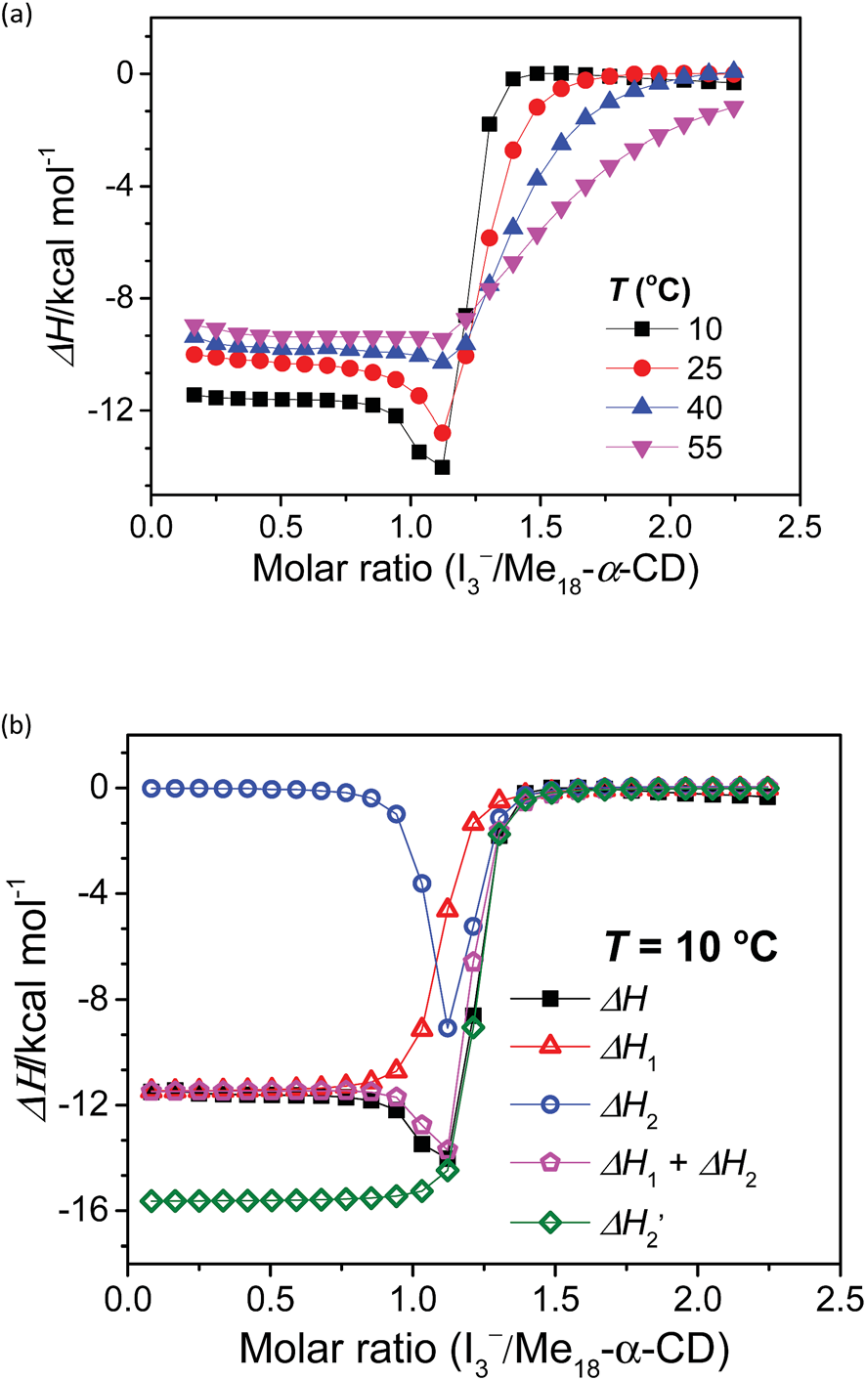
(a) ITC curves of the aqueous solutions of I_3_^–^ to Me_18_-α-CD at various temperatures. (b) Optimum fitting Δ*H*_1_ (red triangles), Δ*H*_2_ (blue circles), sum of Δ*H*_1_ and Δ*H*_2_ (pink pentagons) and experimental result (black squares) of I_3_^–^/Me_18_-α-CD titration at 10 °C. 
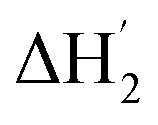
 (green diamonds) is a hypothetical ITC curve for the second binding stage generated by the SSIS model with the absence of the initial binding stage. The enthalpy change in the figures is normalized.

The stoichiometry of Me_18_-α-CD and the polyiodide anion was investigated by isothermal titration calorimetry (ITC) (Fig. 3a). In the case of pristine α-CD, the inflection point of the ITC curve for α-CD with I_3_^–^ was observed at *ca.* 1 : 1, which reflects the 1 : 1 complexation between I_3_^–^ and α-CD.^18^,^24^–^26^ In contrast, the inflection point of the ITC curve for Me_18_-α-CD with I_3_^–^ was *ca.* 1.2 : 1 at 10 °C, which further shifted to 1.3 : 1 with an increase in temperature. This result shows that the interaction of two I_3_^–^ molecules with one Me_18_-α-CD is involved. The most probable reaction is the encapsulation of the I_5_^–^ ion according to the eqn (1) and (2). The formation of I_5_^–^ ions in aqueous Me_18_-α-CD is supported by the UV-Vis and Raman spectral measurements described later.

1 Me_18_-α-CD + I_3_^–^ ⇌ Me_18_-α-CD–I_3_^–^

2 Me_18_-α-CD–I_3_^–^ + I_3_^–^ ⇌ Me_18_-α-CD–I_5_^–^ + I^–^

Since eqn (2) is an equilibrium reaction, the encapsulation reaction occurs at a high concentration of I_3_^–^. The ITC curves in Fig. 3a exhibit a hump of Δ*H* at the molar ratio between 0.8 to 1.1 eq., which can be attributed to the encapsulation of I_5_^–^.

To gain insight into the unique ITC data, curve fitting was executed. In the case of pristine α-CD, the ITC curve could be fitted with a simple 1 : 1 binding model between the host and I_3_^–^ (a simple set of identical sites, SSIS), as reported previously.^18^,^24^–^26^ In contrast, the SSIS model did not fit the ITC curve obtained for the Me_18_-α-CD–I^–^/I_3_^–^ system (Fig. 3a). The fitting was not satisfactory even by applying the TSIS (two sets of independent sites) model, in which two types of sites bind independently to the guest molecules. These observations indicate that the second binding reaction in eqn (2) occurs cooperatively with the complexation between I_3_^–^ and Me_18_-α-CD (eqn (1)). We therefore fitted the ITC curve to the model reported by Kataoka *et al.*, which is a modified model based on the SSIS.^27^ In this model, the concentration of the substance in the second reaction is generated by the initial reaction, and the experimental curve in Fig. 3a was fitted by the simultaneous control of these two interactions. The details of the fitting are revealed in the ESI.† As shown in Fig. 3b, the experimental ITC curve was successively fitted by the sum of Δ*H*_1_ and Δ*H*_2_ derived from the enthalpy changes according to eqn (1) and (2), respectively. The thermodynamic parameters are shown in Table S3.† The ITC curve of the initial binding stage, *i.e.*, I_3_^–^ into Me_18_-α-CD is presented in the red triangles in Fig. 3b. The shift in the stoichiometry from 1 : 1 to *ca.* 1.2 : 1 for the initial bonding stage should be attributed to the contribution of the second binding stage, which consumed a fraction of added I_3_^–^.

**Fig. 4:**
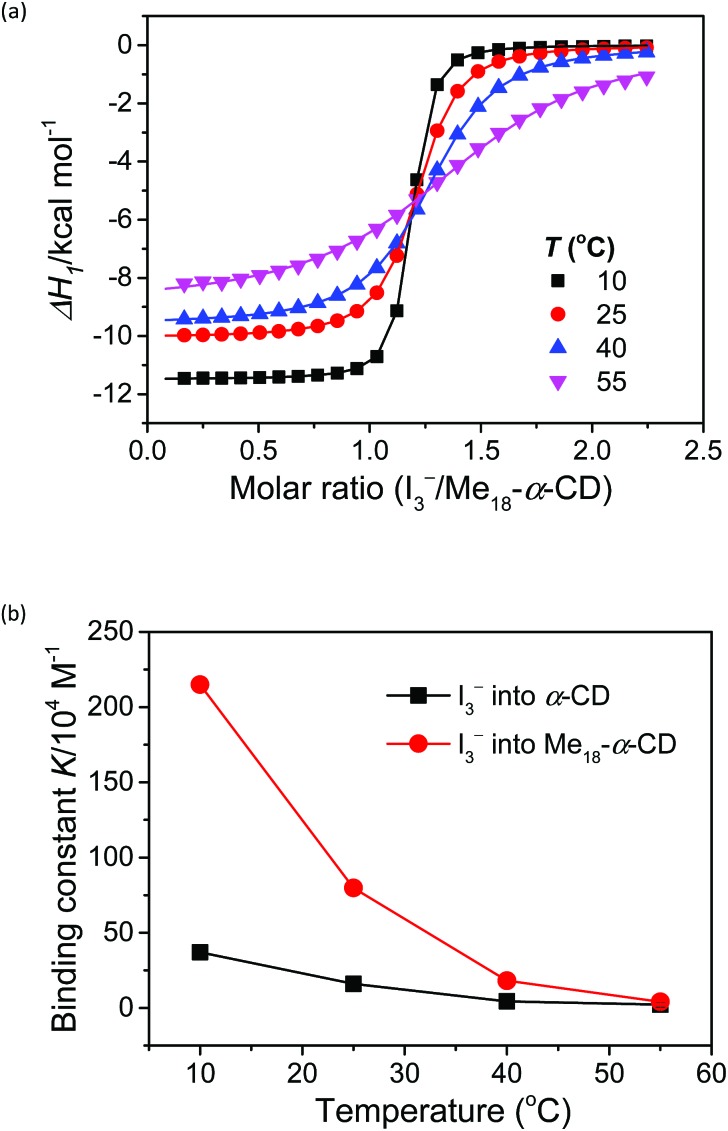
(a) Initial binding enthalpy (Δ*H*_1_ in Fig. 3b) of the ITC curves for Me_18_-α-CD with I_3_^–^ at various temperatures. (b) Temperature difference in the binding constant, *K*, observed for α-CD (black squares) and Me_18_-α-CD (red circles) with I_3_^–^.

The complexation enthalpy between Me_18_-α-CD and I_3_^–^ at various temperatures was extracted in Fig. 4a. The binding constants (*K*) of Me_18_-α-CD and I_3_^–^ were estimated by these fittings and plotted in Fig. 4b. The *K* of pristine α-CD was also plotted in the figure, which is *ca.* 25 times larger at 10 °C than that observed at 55 °C, as reported previously.^18^ On the other hand, Me_18_-α-CD showed remarkable temperature dependence and a *ca.* 50-fold increase in *K* value was observed upon cooling the temperature from 55 °C to 10 °C. This larger change in *K* observed for Me_18_-α-CD and I_3_^–^ enlarged the concentration difference of free I_3_^–^ between the cold- and hot-branches of the cell.

The enhancement in *S*_e_ can be attributed to the changes in association enthalpy, where an increase of –1 kcal mol^–1^ in Δ*H* increases the *S*_e_ by 0.06 mV K^–1^. From Table S3,† the initial binding enthalpy Δ*H*_1_ at 25 °C was –10.1 kcal mol^–1^ and the increment in *S*_e_ was estimated to be 0.6 mV K^–1^. On the other hand, Me_18_-α-CD enhanced the *S*_e_ of the thermocell to 1.08 mV K^–1^. This discrepancy indicates that the association of I_5_^–^ with Me_18_-α-CD further increased the concentration difference of I_3_^–^ between both branches of the cell, which further enhanced the Seebeck coefficient.

### UV-vis spectroscopy

**Fig. 5:**
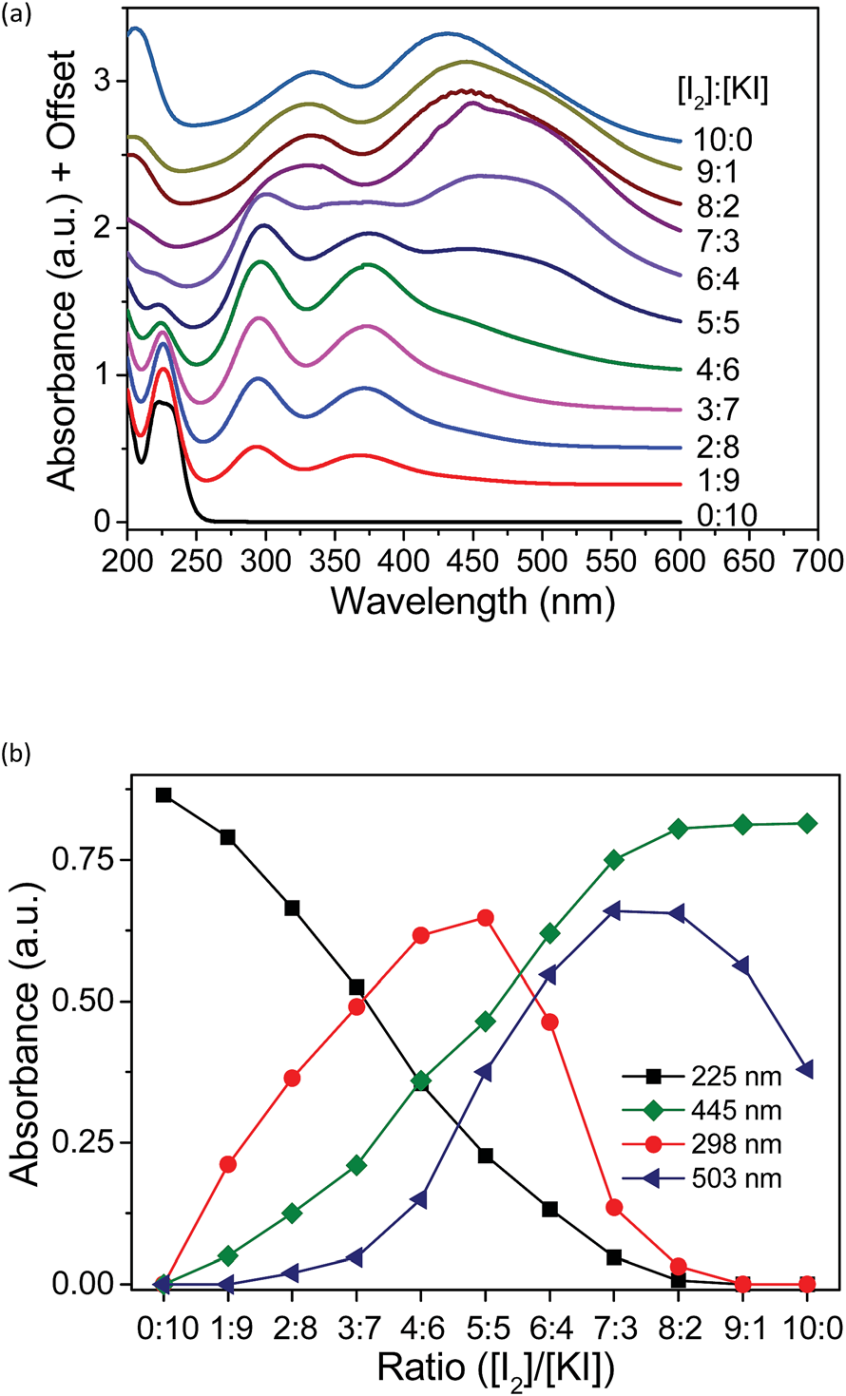
(a) UV-vis spectra of aqueous solutions with varying concentrations. (b) Absorbance of all the peaks in the UV spectra plotted against the ratio of [I_2_]/[KI]. [Me_18_-α-CD] = 2.1 mM.

To investigate the species present in the mixed solution of I_2_, KI and methyl-α-CD, UV-vis spectroscopy was executed (Fig. 5). The peaks were primarily assigned using reference solutions (Fig. S5†). As shown in Fig. S5,† an aqueous solution of pure KI has two peaks at 192 and 225 nm, which are attributed to the absorption peaks of I^–^.^28^,^29^ When I_2_ was added to the KI solution, new peaks emerged at 290 and 352 nm, which reflect the formation of I_3_^–^ ions through eqn (2).^28^–^32^

3 I_2_ + I^–^ ⇌ I_3_^–^

A saturated I_2_ solution without KI has four peaks at 205, 290, 352, and 460 nm, among which, the peaks at 205 and 460 nm are attributed to I_2_.^28^,^29^,^31^,^32^ The other two peaks at 290 and 352 nm are derived from the I_3_^–^ ion generated by the hydrolysis of iodine, as described in the literature.^33^–^35^ The α-CD–I_3_^–^ complex formed upon the addition of α-CD to the aqueous mixture of KI and I_2_ gave almost identical peaks at 290 and 353 nm.^25^,^26^,^35^ Moreover, upon the addition of Me_18_-α-CD, considerable red-shifts to 300 and 375 nm were observed (Fig. S5†), reflecting the complexation by Me_18_-α-CD according to eqn (1). This red-shift in comparison with that of α-CD can be ascribed to the modified bond distance of I_3_^–^ in the relatively deeper and more hydrophobic cavity of Me_18_-α-CD, which may have affected the electronic structure of I_3_^–^.^36^ The component observed for the mixture of I_2_ and Me_18_-α-CD at 430 nm is attributed to Me_18_-α-CD–I_2_, which is blue shifted due to the elevated LUMO level of I_2_ by the interaction with the oxygen atoms of Me_18_-α-CD.^37^–^40^ Furthermore, a shoulder peak at 503 nm was observed in the spectrum (Fig. S5†), which has never been reported for the previous solution systems. In the solid state, the absorption at around 500 nm has been assigned to that of the I_5_^–^ anion;^41^–^46^ thus, we attribute the peak at 503 nm to Me_18_-α-CD–I_5_^–^. The complex is formed through eqn (2).

2 Me_18_-α-CD–I_3_^–^ + I_3_^–^ ⇌ Me_18_-α-CD–I_5_^–^ + I^–^

The formation of Me_18_-α-CD–I_5_^–^ was further confirmed by Raman spectroscopy (Fig. S10†) *via* the increase in the I_2_ stretching signal at *ca.* 170 cm^–1^, which corresponds to I_5_^–^ as an adduct of I^–^·2I_2_.^41^,^47^–^49^

As described above, Me_18_-α-CD provides deeper hydrophobic cavity compared with that of α-CD (Fig. S1†), which must have stabilized the I_5_^–^ species in the form of an Me_18_-α-CD–I_5_^–^ complex. The assignment of the six peaks is summarized in Table S4.† The I_5_^–^ ion has been found in solid polarizer films for liquid crystal displays, but to date, it has not been identified in aqueous solutions. Thus, the formation of Me_18_-α-CD–I_5_^–^ in aqueous solution provides a way to investigate the property of the discrete I_5_^–^ ion.

The existing six peaks were further analyzed using Job's method.^50^ The peaks in Fig. 5a correspond to I^–^ (225 nm), Me_18_-α-CD–I_3_^–^ (298 and 372 nm), Me_18_-α-CD–I_2_ (326 and 445 nm) and Me_18_-α-CD–I_5_^–^ (503 nm). These peaks were separated using Gaussian fitting and the peak intensities were plotted against the initial concentration ratio of I_2_ and KI, as shown in Fig. 5b. The peak intensity of I^–^ at 225 nm monotonically decreased with an increase in the concentration ratio. Moreover, the absorbance of Me_18_-α-CD–I_3_^–^ at 298 nm increased almost proportionally at a low concentration of I_2_. After reaching the maximum intensity at the ratio of 5 : 5, the peak at 298 nm decreased beyond that ratio. The peak at 445 nm (Me_18_-α-CD–I_2_) naturally showed a monotonic increase with an increase in I_2_ concentration. The peak at 503 nm (Me_18_-α-CD–I_5_^–^) showed the maximum at the [I_2_]/[KI] ratio of 7 : 3 to 8 : 2, and decreased beyond this ratio.

If the triiodide is the only polyiodide product in the aqueous mixture of KI and I_2_, the plot of triiodide species (at 298 nm) should give a parabolic curve. The sharp decrease in Me_18_-α-CD–I_3_^–^ (298 nm) and increase in the Me_18_-α-CD–I_5_^–^ (503 nm) species between the ratio of 5 : 5 to 7 : 3 indicated that when the ratio of I_2_ is higher than 5 : 5, Me_18_-α-CD–I_3_^–^ reacts with I_3_^–^ to give the Me_18_-α-CD–I_5_^–^ complex through eqn (2).

Although molecular iodine is slightly soluble in water (1.18 mM, 20 °C),^51^–^53^ no precipitation was observed in our experiments, even in the absence of added KI. This is attributed to the high water solubility of Me_18_-α-CD–I_2_ and formation of Me_18_-α-CD–I_5_^–^. In addition, the weak acidity of the saturated iodine solution of pH 6.6 can be associated with the hydrolysis of iodine. The appearance of each solution is shown in Fig. S7.† The dark color of the 10 : 0 solution indicates the coexistence of Me_18_-α-CD–I_2_ and Me_18_-α-CD–I_5_^–^.

**Fig. 6:**
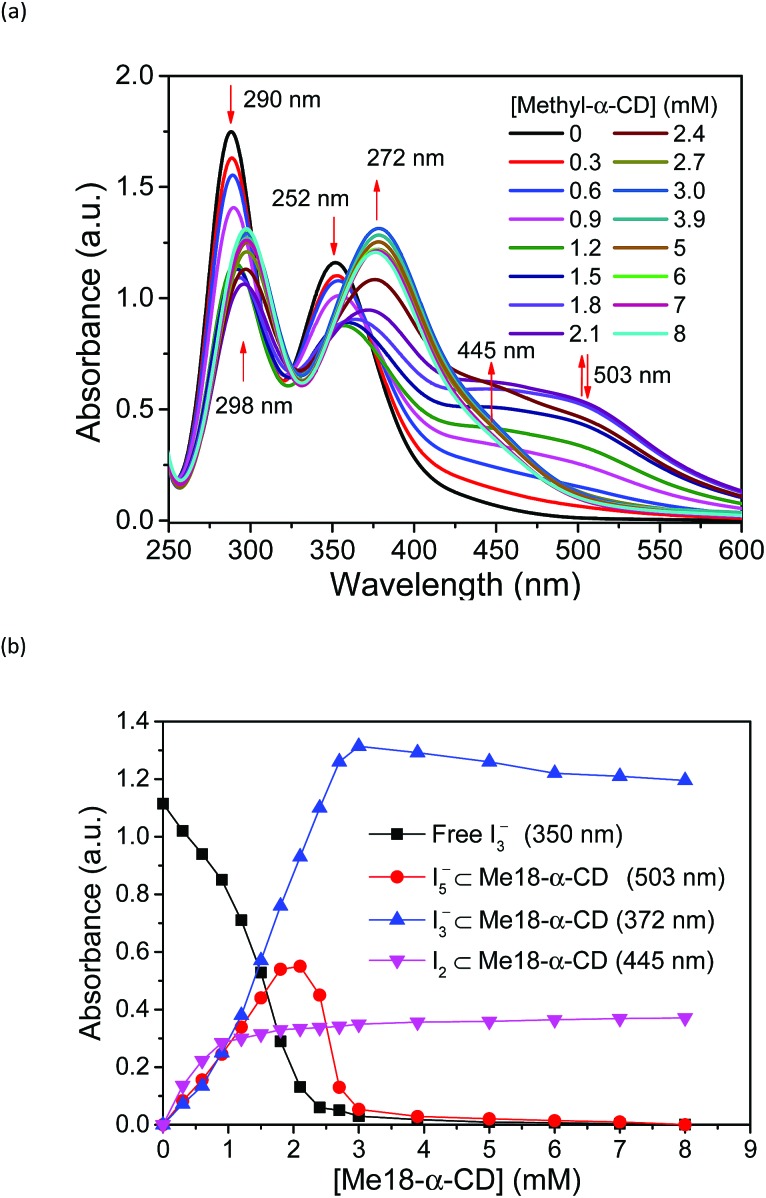
(a) UV-vis spectra of solution 1 with various concentrations of Me_18_-α-CD at 25 °C. (b) Plots of the peak absorbance of I_3_^–^, Me_18_-α-CD–I_3_^–^, Me_18_-α-CD–I_5_^–^, and Me_18_-α-CD–I_2_ in (a).

Based on these spectral analyses, the concentration of the iodide species in the thermocell was then estimated by UV-vis spectroscopy. Fig. 6a shows the UV spectra with various concentrations of Me_18_-α-CD at 25 °C. As discussed previously, the two peaks of I_3_^–^ at 290 and 352 nm showed a red shift upon the addition of Me_18_-α-CD, which corresponds to the formation of Me_18_-α-CD–I_3_^–^. The other peaks at 445 and 503 nm are attributed to Me_18_-α-CD–I_2_ and Me_18_-α-CD–I_5_^–^, respectively. Upon increasing the concentration of Me_18_-α-CD, the intensity of the peaks at 445 and 503 nm increased and the color of the solution changed from yellow to deep red-black (Fig. S6†), which reflect the formation of Me_18_-α-CD–I_5_^–^. Upon the addition of Me_18_-α-CD at higher concentrations beyond 3 mM, the color of the solution returned to weak red (Fig. S6†). To understand these color changes, the intensity of the peaks was plotted in Fig. 6b with a variation in the concentration of the host. As the absorbance of I_3_^–^ (290 nm) decreased, the absorption intensities of the Me_18_-α-CD–I_3_^–^ (372 nm) and Me_18_-α-CD–I_2_ (445 nm) species increased. The absorbance of Me_18_-α-CD–I_3_^–^ and Me_18_-α-CD–I_2_ reached almost constant when the concentration of Me_18_-α-CD was elevated to *ca.* 3 mM. The absorbance of Me_18_-α-CD–I_5_^–^ increased similarly to that of Me_18_-α-CD–I_3_^–^ below the Me_18_-α-CD concentration of 2.1 mM, but it showed an abrupt decrease beyond this host concentration. This indicates that Me_18_-α-CD–I_5_^–^ underwent comproportionation to two Me_18_-α-CD–I_3_^–^ molecules according to eqn (4).^54^–^57^

4 Me_18_-α-CD–I_5_^–^ + Me_18_-α-CD + I^–^ ⇌ 2Me_18_-α-CD–I_3_^–^

**Fig. 7:**
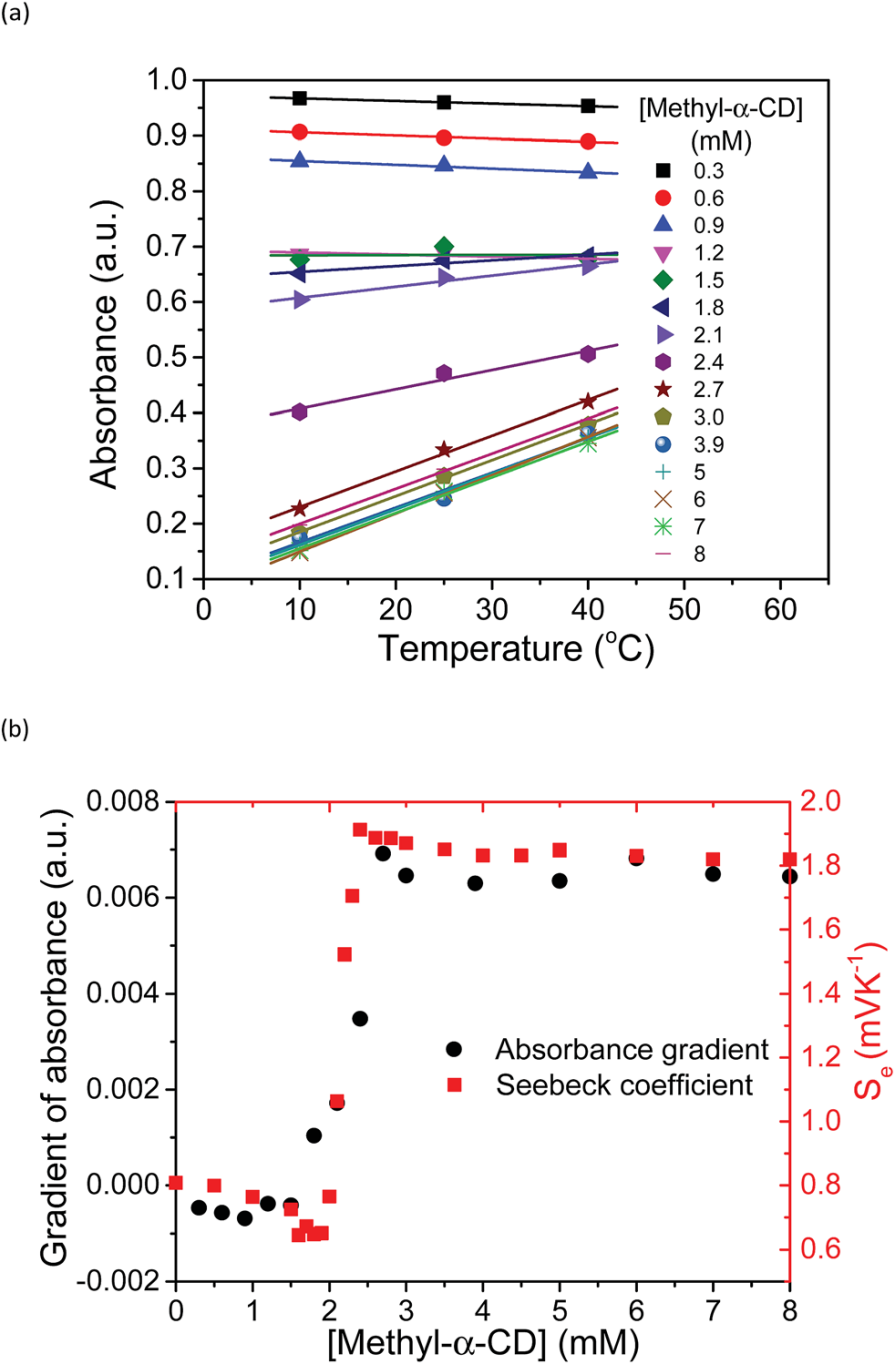
(a) Temperature dependence of the absorbance of free I_3_^–^ species at 352 nm at various concentrations of Me_18_-α-CD at [KI] = 12.5 mM and [I_2_] = 2.5. The slope of the graph changes from negative to positive. (b) Temperature gradient of the UV peak at 352 nm and *S*_e_ at various concentrations of Me_18_-α-CD, where they are in good agreement with each other.

The increase in the *V*_OC_ and *S*_e_ values of the thermocell may be associated with the concentration difference in free I_3_^–^ ions between the lower- and higher-temperature half-cells, which undergo a reduction reaction in the thermocell.^18^ Therefore, the temperature dependence of the concentration of free I_3_^–^ was estimated by UV-vis spectroscopy under various host concentrations. All the spectra are revealed in Fig. S8,† and the peak intensity of free I_3_^–^ species at 352 nm was estimated and plotted at various temperatures and Me_18_-α-CD concentrations, as shown in Fig. 7a. At lower concentrations of Me_18_-α-CD, a slight decrease in the peak intensity was observed with an increase in temperature. The peak intensity of free I_3_^–^ also decreased with an increase in the concentration of Me_18_-α-CD. Upon an increase in temperature, a relatively large increase in the free I_3_^–^ signal was observed for the aqueous Me_18_-α-CD above the host concentration of 1.8 mM. This is ascribed to the decrease in the binding constant between Me_18_-α-CD and I_3_^–^ and the dissociation of the complex to liberate free I_3_^–^ species by heating.

The slope of the lines in Fig. 7a at various Me_18_-α-CD concentrations was plotted in Fig. 7b. The change in the slope drastically increased in the concentration range of 2.0 to 2.2 mM. The change in the I_3_^–^ concentration affected the *S*_e_, and the trend of the graph quite resembles the curve of *S*_e_. Thus, the enhancement of *S*_e_ of the thermocell can be attributed to the host-guest interaction between Me_18_-α-CD and I_3_^–^.

### Temporal stability of the thermocell

**Fig. 8:**
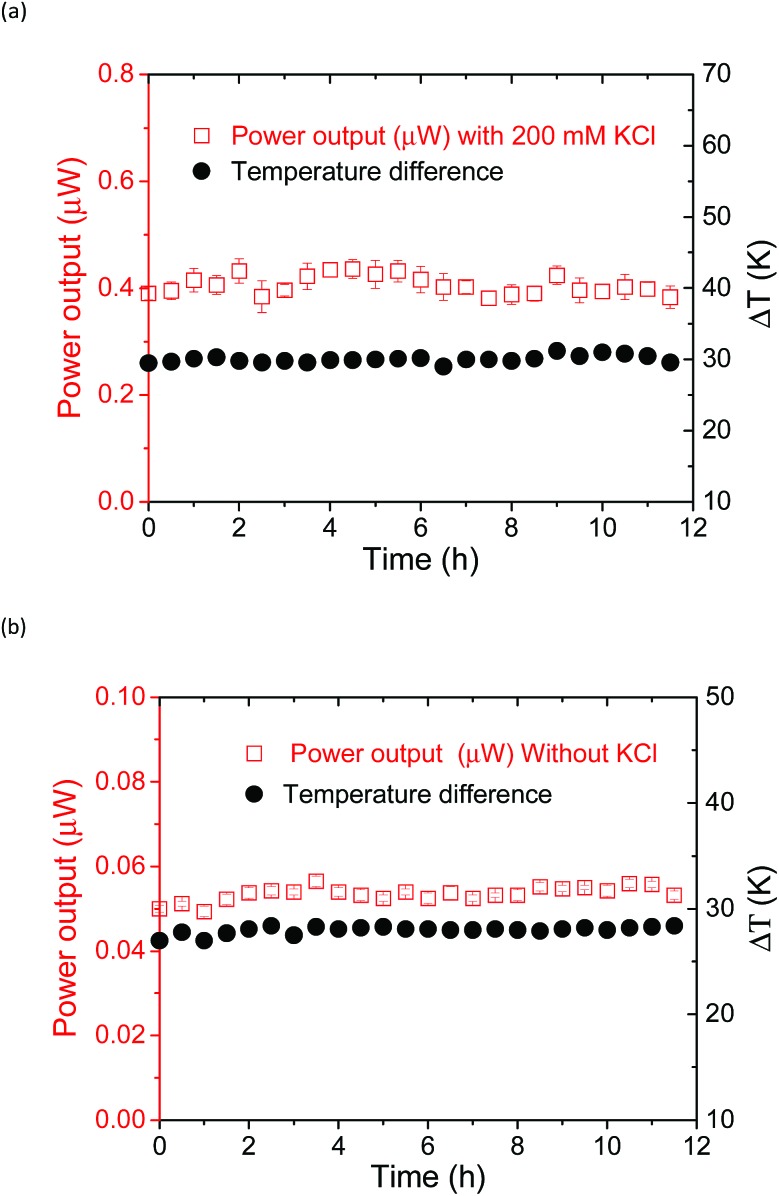
Time dependence of the power output of the thermocell (KI = 12.5 mM, *I*_2_ = 2.5 mM and Me_18_-α-CD = 4 mM) with (a) and without (b) KCl. The temperature difference was controlled at *ca.* 29 K (a) and 30 K (b), which was monitored and added to these figures.

The power output could be obtained by applying an external load voltage (*V*) to the thermocell (Fig. S11†) and measuring the *I*–*V* curves. The maximum power output at each condition was obtained from the plot of power and voltage (Fig. S11b†). The addition of the supporting electrolyte, KCl, to the supramolecular thermocell led to an increase in the current output, as reported previously.^18^ However, in the previous study, precipitation emerged when pristine α-CD was used as the host matrix, and the power output drastically degraded in 6 h. This precipitation occurred due to the hydrogen bonding between the α-CD–I_3_^–^ species, which is the fatal flaw for the long-term operation of α-CD-based thermocells. However, such precipitation was not observed when KCl was added to the aqueous mixture of Me_18_-α-CD, I_2_ and KI. Apparently, methylation of the hydroxy groups effectively prevented precipitation. Fig. 8 shows the time dependence of the power output obtained for the Me_18_-α-CD-based supramolecular thermocell with and without KCl. As shown in this figure, a stable power output was observed, reflecting the stability of the Me_18_-α-CD–I_3_^–^ complex in aqueous media. The stable power output of the Me_18_-α-CD–I_3_^–^-based thermocell, normalized by the temperature difference, was 0.21 mW m^–2^ K^–1^, which is *ca.* 2.3 times higher than that of the α-CD–I_3_^–^-based thermocell after continuous operation for 6 h (0.09 mW m^–2^ K^–1^).

## Conclusions

Me_18_-α-CD was added to an I^–^/I_3_^–^ thermocell and its Seebeck coefficient improved due to host–guest interactions. Compared with pristine α-CD, Me_18_-α-CD showed stronger interaction with I_3_^–^, and the *S*_e_ of the thermocell was enhanced up to 1.9 mV K^–1^ without precipitation. The observed *S*_e_ value is the highest reported for pure-water thermocell systems to date.

UV-vis spectroscopy revealed that I_5_^–^ was captured in the aqueous Me_18_-α-CD, in addition to Me_18_-α-CD–I_3_^–^. This result is the first observation of I_5_^–^ formed in an aqueous system. The binding constants of Me_18_-α-CD and I_3_^–^ were estimated by ITC measurement. The estimated enhancement of *S*_e_ was *ca.* 0.6 mV K^–1^. The additional enhancement of *S*_e_ was derived from the formation of I_5_^–^, which boosted the concentration difference between the hot and cold branches of the cell. The thermal change in the free I_3_^–^ concentration was evaluated by UV-vis measurement, which resembled the *S*_e_ trend. The methylation of the hydroxyl groups in Me_18_-α-CD effectively prevented the formation of the hydrogen-bonded polymer complexes observed for the α-CD–I_3_^–^ complex. As a result, the absence of precipitation in the present Me_18_-α-CD/I_3_^–^ system offered high durability, which was a critical issue in the previous α-CD–I_3_^–^ system. These results indicate that the precise design of the host–guest interaction is imperative to improve the performance of thermocells.

## Conflicts of interest

There are no conflicts to declare.

## Supplementary Material

Supplementary information

## References

[cit1] Biswas K., He J., Blum I. D., Wu C.-I., Hogan T. P., Seidman D. N., Dravid V. P., Kanatzidis M. G. (2012). Nature.

[cit2] Goncalves L. M., Alpuim P., Correia J. H. (2010). J. Electron. Mater..

[cit3] Siddique A. R. M., Mahmud S., Van Heyst B. (2017). Renewable Sustainable Energy Rev..

[cit4] Harman T. C., Walsh M. P., Laforge B. E., Turner G. W. (2005). J. Electron. Mater..

[cit5] Jeffrey Snyder G., Toberer E. S. (2008). Nat. Mater..

[cit6] Quickenden T. I., Mua Y. (1995). J. Electrochem. Soc..

[cit7] Gunawan A., Lin C. H., Buttry D. A., Mujica V., Taylor R. A., Prasher R. S., Phelan P. E. (2013). Nanoscale Microscale Thermophys. Eng..

[cit8] Zhang L., Kim T., Li N., Kang T. J., Chen J., Pringle J. M., Zhang M., Kazim A. H., Fang S., Haines C., Al-Masri D., Cola B. A., Razal J. M., Di J., Beirne S., MacFarlane D. R., Gonzalez-Martin A., Mathew S., Kim Y. H., Wallace G., Baughman R. H. (2017). Adv. Mater..

[cit9] Mua Y., Quickenden T. I. (1996). J. Electrochem. Soc..

[cit10] Quickenden T. I., Vernon C. F. (1986). Sol. Energy.

[cit11] Dupont M. F., MacFarlane D. R., Pringle J. M. (2017). Chem. Commun..

[cit12] Hu R., Cola B. A., Haram N., Barisci J. N., Lee S., Stoughton S., Wallace G., Too C., Thomas M., Gestos A., Dela Cruz M. E., Ferraris J. P., Zakhidov A. A., Baughman R. H. (2010). Nano Lett..

[cit13] Im H., Kim T., Song H., Choi J., Park J. S., Ovalle-Robles R., Yang H. D., Kihm K. D., Baughman R. H., Lee H. H., Kang T. J., Kim Y. H. (2016). Nat. Commun..

[cit14] Romano M. S., Li N., Antiohos D., Razal J. M., Nattestad A., Beirne S., Fang S., Chen Y., Jalili R., Wallace G. G., Baughman R., Chen J. (2013). Adv. Mater..

[cit15] Abraham T. J., MacFarlane D. R., Pringle J. M. (2013). Energy Environ. Sci..

[cit16] Abraham T. J., MacFarlane D. R., Pringle J. M. (2011). Chem. Commun..

[cit17] Kim T., Lee J. S., Lee G., Yoon H., Yoon J., Kang T. J., Kim Y. H. (2017). Nano Energy.

[cit18] Zhou H., Yamada T., Kimizuka N. (2016). J. Am. Chem. Soc..

[cit19] Zhou H., Yamada T., Kimizuka N. (2018). Sustainable Energy Fuels.

[cit20] Nour E. M., Chen L. H., Laane J. (1986). J. Phys. Chem..

[cit21] Havinga E. E., Wiebenga E. H. (1958). Acta Crystallogr..

[cit22] Hach R. J., Rundle R. E. (1951). J. Am. Chem. Soc..

[cit23] Herbstein F. H., Kapon M. (1972). Nature Phys. Sci..

[cit24] Kitamura S., Nakatani K., Takaha T., Okada S. (1999). Macromol. Rapid Commun..

[cit25] Pursell J. L., Pursell C. J. (2016). J. Phys. Chem. A.

[cit26] Minns J. W., Khan A. (2002). J. Phys. Chem. A.

[cit27] Kim W., Yamasaki Y., Kataoka K. (2006). J. Phys. Chem. B.

[cit28] Kireev S. V., Shnyrev S. L. (2015). Laser Phys..

[cit29] Wei Y. J., Ge L. C., Mo L. P. (2005). Spectrosc. Spectr. Anal..

[cit30] Bichsel Y., Von Gunten U. (1999). Anal. Chem..

[cit31] Palmer D. A., Ramette R. W., Mesmer R. E. (1984). J. Solution Chem..

[cit32] Troy R. C., Kelley M. D., Nagy J. C., Margerum D. W. (1991). Inorg. Chem..

[cit33] Lengyel I., Epstein I. R., Kusth K. (1993). Inorg. Chem..

[cit34] Minns J. W., Khan A. (2002). J. Phys. Chem. A.

[cit35] Calabrese V., Khan A. (2000). J. Phys. Chem. A.

[cit36] Mould D. L. (1954). Biochem. J..

[cit37] Mulliken R. S. (1950). J. Am. Chem. Soc..

[cit38] Lang R. P. (1962). J. Am. Chem. Soc..

[cit39] Kim K. H., Ki H., Lee J. H., Park S., Kong Q., Kim J., Kim J., Wulff M., Ihee H. (2015). Phys. Chem. Chem. Phys..

[cit40] Reichardt C. (1994). Chem. Rev..

[cit41] Svensson P. H., Kloo L. (2003). Chem. Rev..

[cit42] Kim H. (1982). Biopolymers.

[cit43] Saenger W. (1984). Naturwissenschaften.

[cit44] Wiebenga E. H., Havinga E. E. and Boswijk K. H., Advances inoganic Chem. Radiochem., 1961, vol. 3, pp. 133–169.

[cit45] Mizuno M., Tanaka J., Harada I. (1981). J. Phys. Chem..

[cit46] Noltermeyer M., Saenger W. (1976). Nature.

[cit47] Deplano P., Ferraro J. R., Mercuri M. L., Trogu E. F. (1999). Coord. Chem. Rev..

[cit48] Svensson P. H., Kloo L. (2003). Chem. Rev..

[cit49] Bengtsson L. A., Stegemann H., Holmberg B., Füllbier H. (1991). Mol. Phys..

[cit50] Job P. (1928). Ann. Chim. Appl..

[cit51] Katzin L. I., Gebert E. (1955). J. Am. Chem. Soc..

[cit52] Kracek F. C. (1931). J. Phys. Chem..

[cit53] Diaz D., Vargas-Baca I., Gracia-Mora J. (1994). J. Chem. Educ..

[cit54] Martin L., Yang S., Brooks A. C., Horton P. N., Male L., Moulfi O., Harmand L., Day P., Clegg W., Harrington W. (2015). CrystEngComm.

[cit55] Filgueiras C. A., Horn J. A., Skakle J. M., Wardell J. L. (2001). Acta Crystallogr., Sect. E: Struct. Rep. Online.

[cit56] Blake A. J., Devillanova F. A., Gould R. O., Li W., Parsons S., Schr M. (1998). Chem. Soc. Rev..

[cit57] Kloo L., Svensson H., Taylor M. J. (2000). J. Chem. Soc., Dalton Trans..

