# Supplementary material for: Hexakis(2,3,6-tri-O-methyl)-α-cyclodextrin–I5– complex in aqueous I–/I3– thermocells and enhancement in the Seebeck coefficient
Source: Chem Sci. 2018 Oct 22;10(3):773–80. doi: 10.1039/c8sc03821j (PMC6340408; doi:10.1039/c8sc03821j)
Supplement: Supplementary file 1 [file SC-010-C8SC03821J-s001.pdf]

Electronic Supplementary Material (ESI) for Chemical Science.
This journal is © The Royal Society of Chemistry 2018

Electronic Supplementary Information

Hexakis(2,3,6-tri-*O*-methyl)-*α*-cyclodextrin–I_5^- Complex in Aqueous I^-/I_3^- Thermocells and Enhancement in the Seebeck Coefficient

Yimin Liang,[a] Teppei Yamada,*[a,b] Hongyao Zhou,[a] and Nobuo Kimizuka[a,b]

[a] Division of Chemistry and Biochemistry, Graduate School of Engineering, Kyushu University, 744 Moto-oka, Nishi-ku, Fukuoka 819-0395 (Japan), E-mail: teppei343@gmail.com

[b] Center for Molecular System (CMS), Kyushu University, 744 Moto-oka, Nishi-ku, Fukuoka 819-0395 (Japan)

Table of Contents

1. **Materials and Sources** 2
2. **Experimental** 2
   - 2.1. **Preparation of aqueous solution** 2
   - 2.2. **Seebeck Coefficient Measurements** 4
   - 2.3. **Isothermal Titration Calorimetry (ITC) measurements** 4
     - 2.3.1. **SSIS model** 5
     - 2.3.2. **The fitting of the present research** 6
   - 2.4. **UV-vis spectroscopy** 10
   - 2.5. **Raman spectroscopy** 15
   - 2.6. **Power output measurement of the KI/I2/M18-*α*-CD thermocell** 17

References 18

1. Materials and Sources

Iodine (99.8%), Potassium iodide (99.5%) and Potassium chloride (>99%) were purchased from Kanto Chemical Co. Inc. (Japan). *α*-Cyclodextrin (*α*-CD) and hexakis(2,3,6-*O*-Me)-*α*-cyclodextrin (Me_{18}-*α*-CD) were purchased from Cyclodextrin-Shop of AraChem (Tilburg, Netherlands). All reagents were used without any further purification.

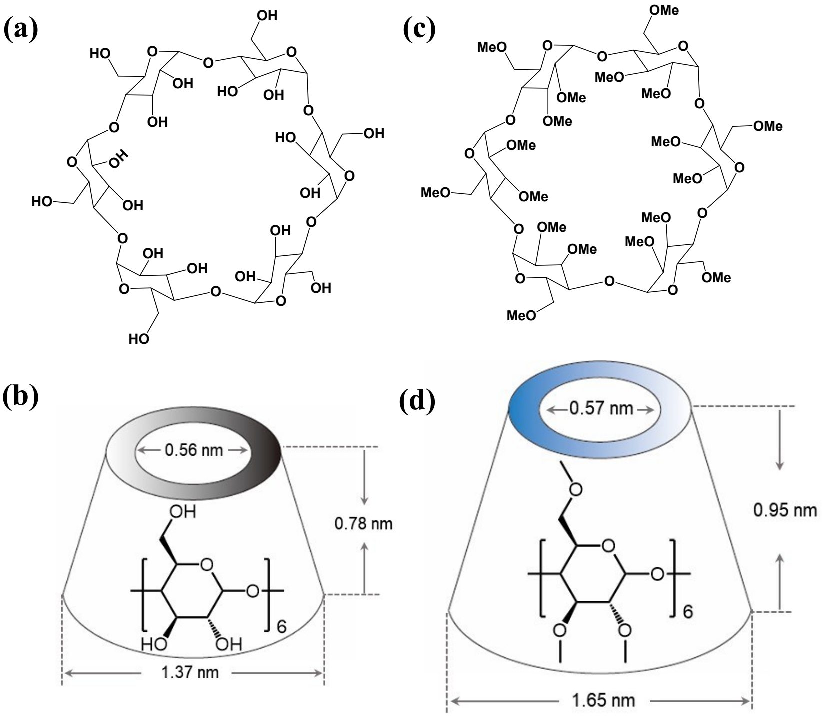

Figure S1. (a, b) The molecular structure and size of *α*-Cyclodextrin (*α*-CD)[1] and (c, d) hexakis(2,3,6-tri-*O*-methyl)-*α*-Cyclodextrin (Me_{18}-*α*-CD)[2], respectively.

2. Experimental

2.1. Preparation of aqueous solution

Various kinds of stock solutions were prepared as summarized in Table S1 and Table S2.

Solution 1: I_2 (25.4 mg, 1.0 mmol), KI (83 mg, 5.0 mmol) and various amount of Me_{18}-*α*-CD were dissolved into 40 ml water so that initial concentration of KI and I_2 to be 12.5 and 2.5 mM, respectively. The concentration of Me_{18}-*α*-CD was listed in Table S1. The solutions were called as S1-x where x corresponds to the concentration of Me_{18}-*α*-CD (mM).

Table S1. The mixing conditions of Solution 1.

	I_2 (mM)	KI (mM)	Me_{18}-α-CD (mM)
**S1-0.0**	2.5	12.5	0
**S1-0.3**	2.5	12.5	0.3
**S1-0.6**	2.5	12.5	0.6
**S1-0.9**	2.5	12.5	0.9
**S1-1.2**	2.5	12.5	1.2
**S1-1.5**	2.5	12.5	1.5
**S1-1.8**	2.5	12.5	1.8
**S1-2.1**	2.5	12.5	2.1
**S1-2.4**	2.5	12.5	2.4
**S1-2.7**	2.5	12.5	2.7
**S1-3.0**	2.5	12.5	3.0
**S1-3.9**	2.5	12.5	3.9
**S1-5.0**	2.5	12.5	5.0
**S1-6.0**	2.5	12.5	6.0
**S1-8.0**	2.5	12.5	8.0

Solution 2: Solutions containing various I_2/KI ratio were prepared. The concentration of each solution was listed in Table S2. These solutions are called S2-a:b where a:b corresponds to the ratio of I_2 and KI.

Table S2 The mixing conditions of Solution 2.

	I_2 (mM)	KI (mM)	Me_{18}-α-CD (mM)
**S2-0:10**	0	3.50	2.10
**S2-1:9**	0.35	3.15	2.10
**S2-2:8**	0.70	2.80	2.10
**S2-3:7**	1.05	2.45	2.10
**S2-4:6**	1.40	2.10	2.10
**S2-5:5**	1.75	1.75	2.10
**S2-6:4**	2.10	1.40	2.10
**S2-7:3**	2.45	1.05	2.10
**S2-8:2**	2.80	0.70	2.10
**S2-9:1**	3.15	0.35	2.10
**S2-10:0**	3.50	0	2.10

2.2. Seebeck Coefficient Measurements

40 ml of Solution 1 (S1-0.0 to S1-8.0) were poured into the H-shaped glass cell consists of two half-cells soaked into a couple of water baths at different temperature (Fig. S2). Each half-cell has a diameter of ca. 2 cm and the distance between them is ca. 10 cm for creating a stable temperature difference. The temperatures inside the half-cells were monitored with thermometers (TM201, AS ONE, Japan) and the cold side was kept at approximately 10 ℃. Meanwhile, the electrolyte was stirred during the measurements to accelerate the equilibrium in the cell. Platinum wires (φ = 1 mm) were washed with concentrated sulfuric acid and used as electrodes for measuring a potential difference generated by the temperature difference. The potential difference was recorded by a source meter 2401 (KEITHLEY).

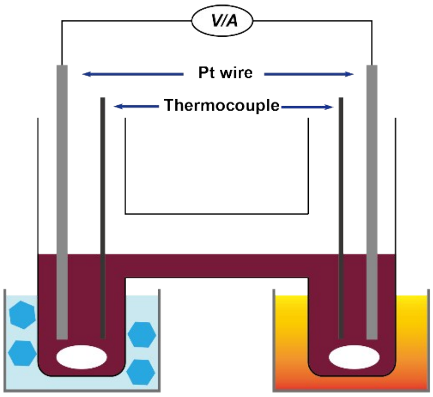

Figure S2. Schematic illustration of the H-shaped cell. The two half-cells were immersed in cold and hot water baths. A stirrer was put into each side to accelerate the thermal equilibrium. Two platinum wire electrodes were used to measure the open circuit voltage (V_{oc}) and the temperature difference was evaluated by thermocouples.

2.3. Isothermal Titration Calorimetry (ITC) measurements

The MicroCal VP-ITC titrator (LLC, Northampton, Ma, USA) was used for the calorimetric titrations. An aqueous solution of 13.5 mM of KI and 3.5 mM of I_2 was previously degassed by means of a vacuum degasser Thermovac (MicroCal, LLC, Northampton, Ma, USA). The solution was drawn into the syringe (250 µl). Another aqueous solution containing 0.3 mM of Me_{18}-α-CD was also degassed and introduced

into the ITC cell (1.4 ml). Data were collected during twenty-five injections of 10 µl I_3^-/I^- titrant into Me_{18}-α-CD titrate (duration 24 s) at 10, 25, 40 and 55 ℃ with stirring at 310 rpm and 240 s injection spacing.

The fitting model is not the general single set of identical sites (SSIS) or two sets of independent sites (TSIS), as discussed in the manuscript. Thus, the experimental results were analyzed by a novel fitting method based on the single set of identical sites (SSIS).

In the novel fitting model, the concentration of the substances in Eq. 2 is dependent on the initial binding reaction (Eq. 1).

Me_{18}-α-CD + I_3^- ⇌ Me_{18}-α-CD–I_3^- (**Eq. 1**)

Me_{18}-α-CD–I_3^- + I_3^- ⇌ Me_{18}-α-CD–I_5^- + I^- (**Eq. 2**)

The total heat generated can be regarded as a sum of Eq. 1 and Eq. 2 and generated heat at each injection can be written as Eq. S1,

$$\Delta Q(i) = \Delta Q(i)_1 + \Delta Q(i)_2 \qquad \textbf{(Eq. S1)}$$

where $\Delta Q(i)$ is the heat generated of the ith injection step, $\Delta Q(i)_1$ and $\Delta Q(i)_2$ are heat generated by Eq. 1 and Eq. 2, respectively.

The reaction of each step can be fitted by SSIS model.

2.3.1. SSIS model

The SSIS model is introduced as follows:[3–5]

K = Binding constant;

n = The number of the binding sites;

V_o = Cell volume;

M_t = The total concentration of host molecules in $V_{o;}$

X_t and [X] are total and free concentrations of guest molecules;

Θ = the fraction of binding sites occupied by a guest.

The binding constant K and the relationship of the total and free guest are described as:

$$K = \frac{\Theta}{(1 - \Theta)[X]} \qquad \textbf{(Eq. S2)}$$

$$X_t = [X] + n\Theta M_t \qquad \textbf{(Eq. S3)}$$

Combing Eq. S1 and Eq. S2 gives

$$\Theta^2 - \Theta\left[1 + \frac{X_t}{nM_t} + \frac{1}{nKM_t}\right] + \frac{X_t}{nM_t} = 0 \qquad \textbf{(Eq. S4)}$$

The total heat content Q of the solution contained in V_o at fractional saturation Θ is

$$Q = n\Theta M_t \Delta H V_o \qquad \textbf{(Eq. S5)}$$

Where ΔH is the molar heat of guest binding. Solving the quadratic equation (Eq. S4) for Θ and then substituting this into Eq. S5 gives

$$Q = \frac{nM_t\Delta HV_o}{2}\left[1+\frac{X_t}{nM_t}+\frac{1}{nKM_t}-\sqrt{(1+\frac{X_t}{nM_t}+\frac{1}{nKM_t})^2-\frac{4X_t}{nM_t}}\right] \qquad \textbf{(Eq. S6)}$$

The value of Q above can be calculated at the end of the ith injection and designated $Q(i)$. The parameter of interest for comparison with experiment, however, is the change in heat content from the completion of the $(i\text{-}1)^{th}$ injection to completion of the ith injection. The expression for Q in Eq. S5 only applies to the liquid contained in volume V_o. Therefore, after completing an injection, it is obvious that a correction must be made for displaced volume (i.e., ΔV_i = injection volume) since some of the liquid in V_o after the $i\text{-}1^{th}$ injection will no longer be in V_o after the ith injection, even though it will contribute to the heat effect (assuming the kinetics of reaction and mixing are fast) before it passes out of the working volume V_o. The liquid in the displaced volume contributes about 50% as much heat effect as an equivalent volume remaining in V_o. The correct expression for the heat released $\Delta Q(i)$, from the ith injection is

$$\Delta Q(i) = Q(i) + \frac{dV_i}{V_o}\left[\frac{Q(i)+Q(i-1)}{2}\right] - Q(i-1) \qquad \text{(Eq. S7)}$$

By dividing $\Delta Q(i)$ with moles in the ith injected volume, the normalized heat, $\Delta Q(i)$ is obtained, which is described by three parameters n, ΔH, and K.

2.3.2. The fitting of the present research

In the current fitting model, the total generated heat can be separated by two kinds of heat $Q_1(i)$ and $Q_2(i)$, which are generated according to Eq. 1, and Eq. 2, respectively.

$$Q_1(i) = \frac{n_1 M_{t1} \Delta H_1 V_0}{2}\left[1 + \frac{X_{t1}}{n_1 M_{t1}} + \frac{1}{n_1 K_1 M_{t1}} - \sqrt{(1 + \frac{X_{t1}}{n_1 M_{t1}} + \frac{1}{n_1 K_1 M_{t1}})^2 - \frac{4X_{t1}}{n_1 M_{t1}}}\right]$$

$$Q_2(i) = \frac{n_2 M_{t2} \Delta H_2 V_0}{2}\left[1 + \frac{X_{t2}}{n_2 M_{t2}} + \frac{1}{n_2 K_2 M_{t2}} - \sqrt{(1 + \frac{X_{t2}}{n_2 M_{t2}} + \frac{1}{n_2 K_2 M_{t2}})^2 - \frac{4X_{t2}}{n_2 M_{t2}}}\right]$$

Where,

ΔH_1 = Enthalpy change at the initial binding stage;

K_1 = Binding constant between I_3^- and Me_{18}-α-CD;

n_1 = The number of the binding sites in Me_{18}-α-CD;

V_o = Cell volume;

M_{t1} = The total concentration of Me_{18}-α-CD in V_o;

X_{t1} = The total concentration of I_3^-;

$Q_1(i)$: The total released heat until the ith injection in the initial stage (Eq. 1).

Similarly, the symbols in the second binding stage can be defined as:

ΔH_2 = The enthalpy change at the second binding stage

K_2 = Equilibrium constant between I_3^- and I_3^-–Me_{18}-α-CD

N_2 = The number of the binding sites in I_3^-–Me_{18}-α-CD

V_o = Cell volume

M_{t2} = The total concentration of I_3^-–Me_{18}-α-CD in V_o, which equals to $X_{t1}[\Delta Q_1/(i\Delta H_1)]$

X_{t2} = The total concentration of I_3^-, which equals to $X_{t1}[1-\Delta Q_1/(i\Delta H_1)]$

$Q_2(i)$: The total released heat until the ith injection in the second stage (Eq. 2).

Then, the experimental ITC curve can be fitted by $\Delta Q = \Delta Q(i)_1 + \Delta Q(i)_2$, where $\Delta Q(i)_1$ and $\Delta Q(i)_2$ are the released heat for i^{th} titration in the initial and second stages, respectively. The result in the thermodynamic parameters ΔH_1, K_1, ΔH_2, and K_2. Are shown in Table S3. ΔS_1 and ΔS_2 were obtained by van't Hoff equation.

Table S3. Parameters for initial and second binding stages.

**Me_{18}-*α*-CD + I_3^- → Me_{18}-*α*-CD–I_3^-**				
	**10 °C**	**25 °C**	**40 °C**	**55 °C**
**ΔH_1 (kcal mol^{-1})**	−11.5	−10.1	−9.61	−8.89 ± 0.04
**ΔS (cal K^{-1} mol^{-1})**	−6.03	−8.15	−9.86	−10.28
**$K_{as}/10^4$ (M^{-1})**	215 ± 5	79.8 ± 0.5	18.1 ± 0.4	4.12 ± 0.13
**Me_{18}-*α*-CD–I_3^-+ I_3^- → Me_{18}-*α*-CD–I_5^- + I^-**				
**ΔH_2 (kcal mol^{-1})**	−15.6	−15.99	−15.39	−17.75
**ΔS (cal K^{-1} mol^{-1})**	−24.38	−29.35	−29.95	−41.64
**$K_{as}/10^4$ (M^{-1})**	564 ± 5	85.1 ± 0.3	21.5±1.0	4.01±0.04

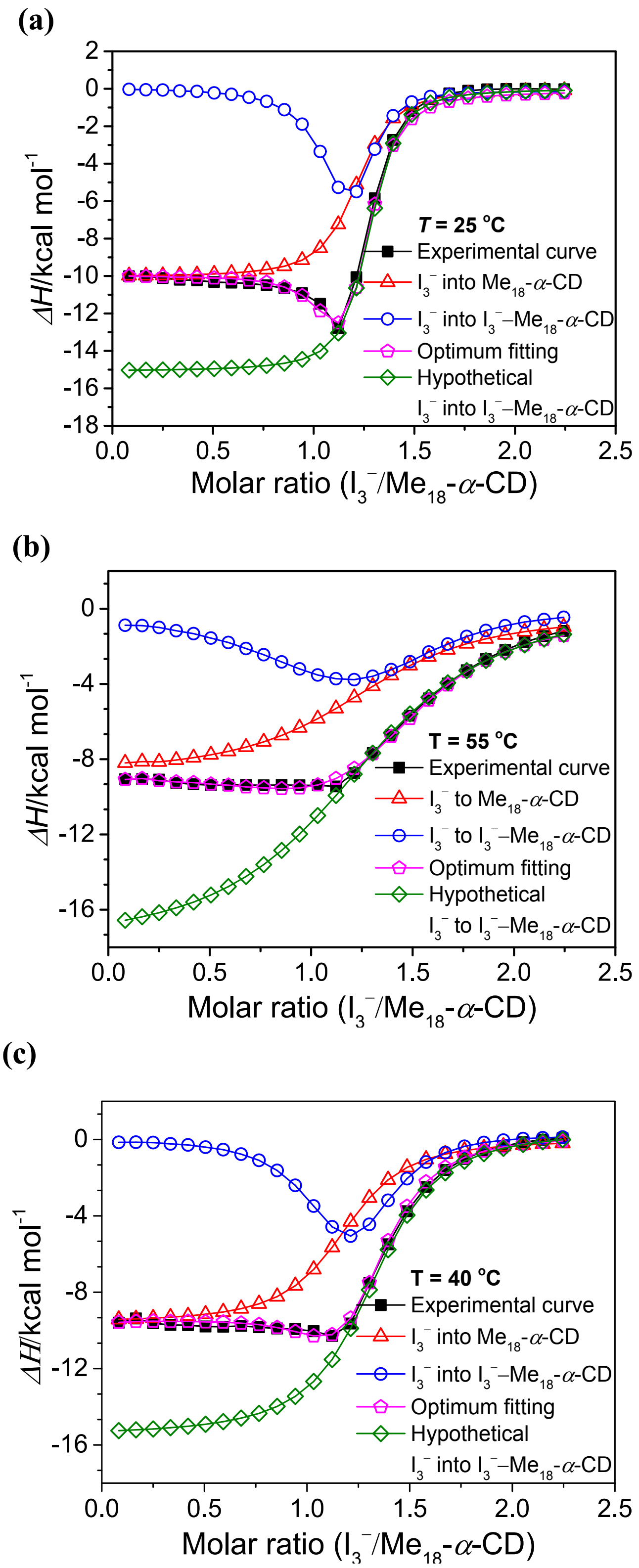

Figure S3. Optimum fitting of the experimental titration curve for the sum of I_3^- into Me_{18}-α-CD and I_3^- into I_3^-–Me_{18}-α-CD.

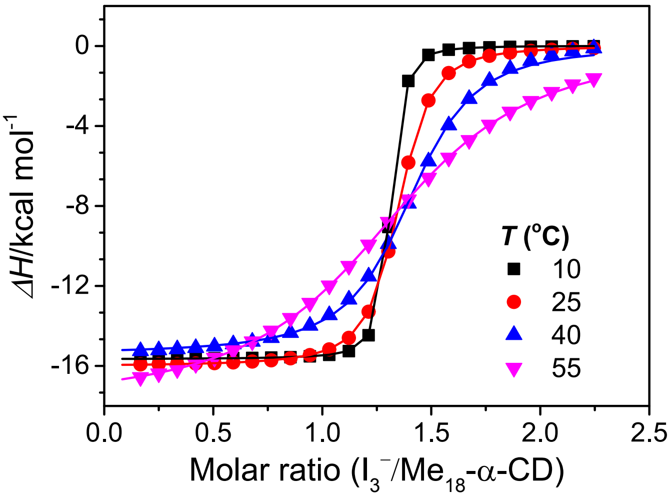

Figure S4 The ITC curves for the second binding stage, i.e., I_3^- into Me_{18}-*α*-CD–I_3^- at various temperatures**.**

2.4. UV-vis spectroscopy

UV-vis spectra were recorded by UV-vis spectrometer (V670, JASCO, Japan) in the wavelength range between 200 to 600 nm, with a resolution of 0.5 nm and a fixed slit width of 0.5 nm. 0.2 mm path length quartz cuvettes were used.

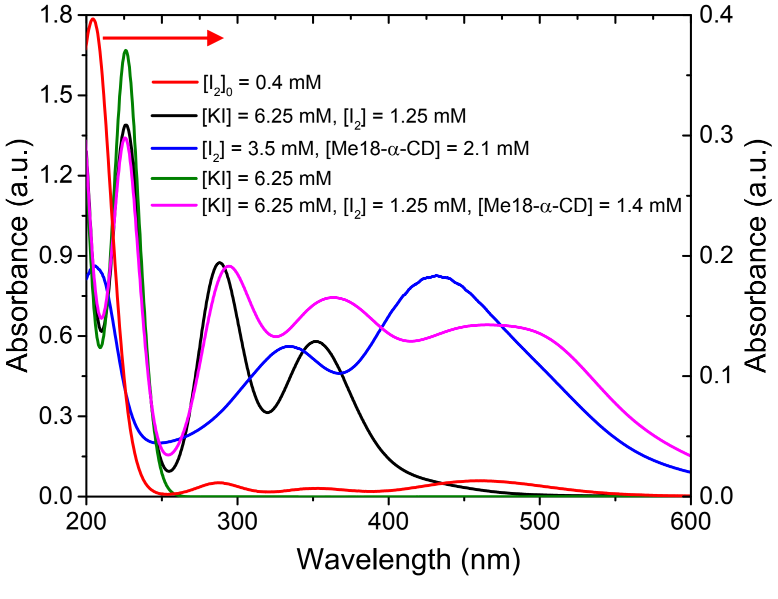

Figure S5. The UV-vis spectra of UV-vis spectra of various aqueous solutions of 6.25 mM of KI (green); 6.5 mM of KI and 1.25 mM of I_2 (black); 0.4 mM of I_2 (red); 6.25 mM of KI, 1.25 mM of I_2 and 1.4 mM of Me18-*α*-CD (pink); and 3.5 mM of I_2 and 2.1 mM of Me18-*α*-CD, which were used to attribute all of the peaks in I_2, KI and Me_{18}-*α*-CD composed solutions.

Table S4. The summary of the attribution of peaks on UV-vis spectra

	I^-	I_2	Me$_{18}$-α-CD–I_2	I_3^-	Me$_{18}$-α-CD–I_3^-	Me$_{18}$-α-CD–I_5^-
**Wavelength (nm)**	192	205	326	290	298	503
	225	460	445	352	372	

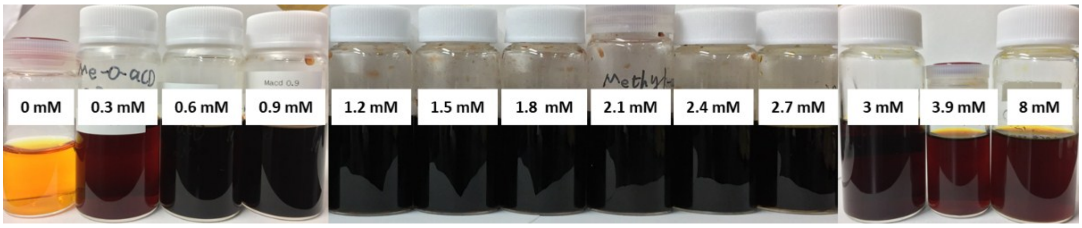

Figure S6. The pictures of Solutions 1 (see Table S1) at varied Me$_{18}$-α-CD concentrations. The color of solutions changes from yellow to red black then to deep red-black. When the concentration of Me$_{18}$-α-CD is increased beyond 3 mM, the color returns to red black again. The change of the color indicates the formation of Me$_{18}$-α-CD–I_5^- and its transformation to Me$_{18}$-α-CD–I_3^- species at higher Me$_{18}$-α-CD concentrations.

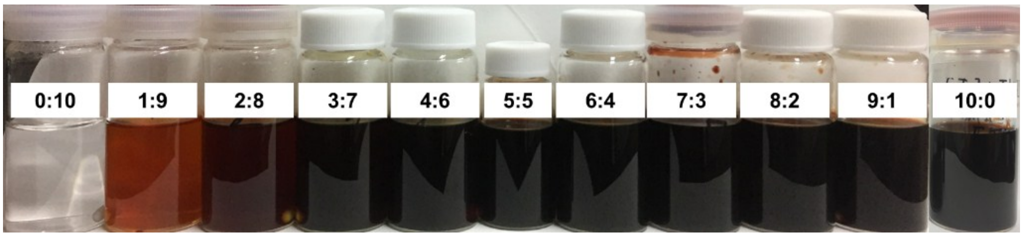

Figure S7. The appearance of Solutions 2 at room temperature. Because of the Me$_{18}$-α-CD inclusion and iodine hydrolysis, an excess of iodine can be dissolved in water even at the high ratio of 10:0.

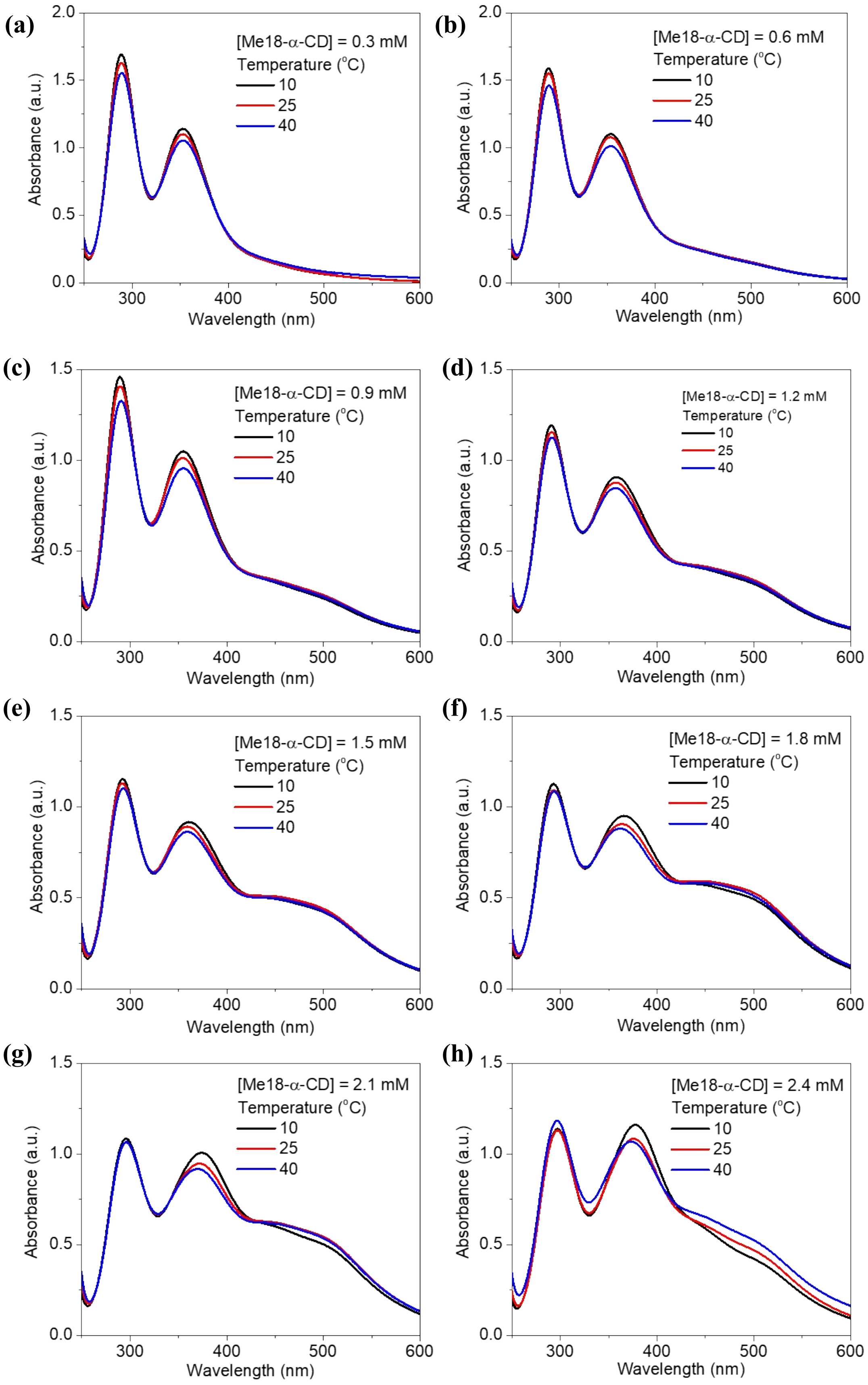
(a)
[Me18-α-CD] = 0.3 mM
Temperature (°C)
10
25
40
Absorbance (a.u.)
Wavelength (nm)
(b)
[Me18-α-CD] = 0.6 mM
Temperature (°C)
10
25
40
Absorbance (a.u.)
Wavelength (nm)
(c)
[Me18-α-CD] = 0.9 mM
Temperature (°C)
10
25
40
Absorbance (a.u.)
Wavelength (nm)
(d)
[Me18-α-CD] = 1.2 mM
Temperature (°C)
10
25
40
Absorbance (a.u.)
Wavelength (nm)
(e)
[Me18-α-CD] = 1.5 mM
Temperature (°C)
10
25
40
Absorbance (a.u.)
Wavelength (nm)
(f)
[Me18-α-CD] = 1.8 mM
Temperature (°C)
10
25
40
Absorbance (a.u.)
Wavelength (nm)
(g)
[Me18-α-CD] = 2.1 mM
Temperature (°C)
10
25
40
Absorbance (a.u.)
Wavelength (nm)
(h)
[Me18-α-CD] = 2.4 mM
Temperature (°C)
10
25
40
Absorbance (a.u.)
Wavelength (nm)

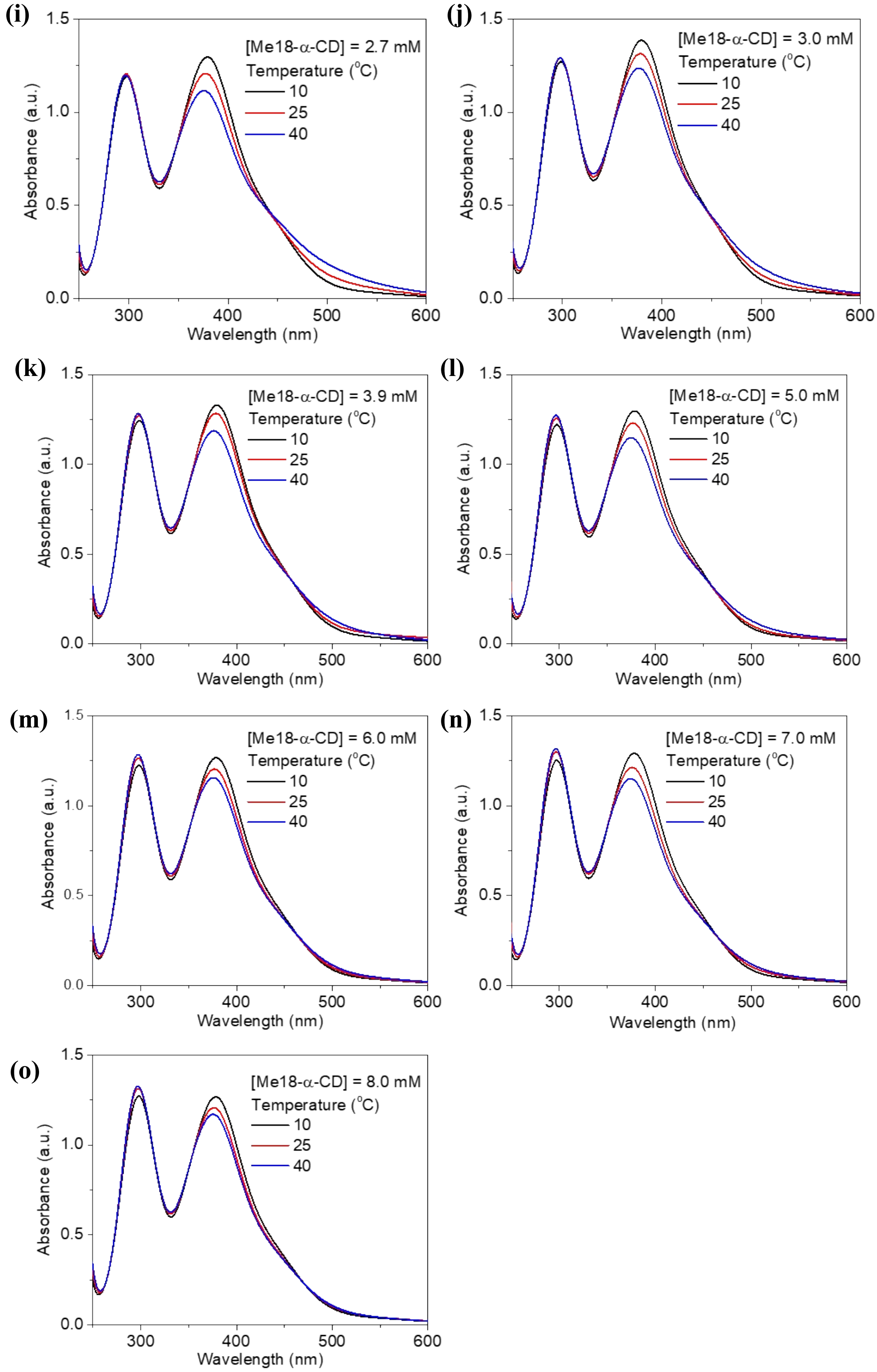

Figure S8. The UV-vis spectra of Solution 1 at 10, 25 and 40 °C.

(a)

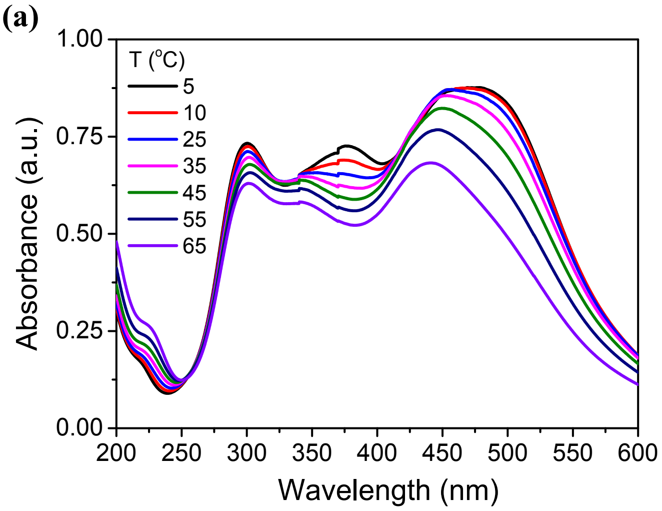

(b)

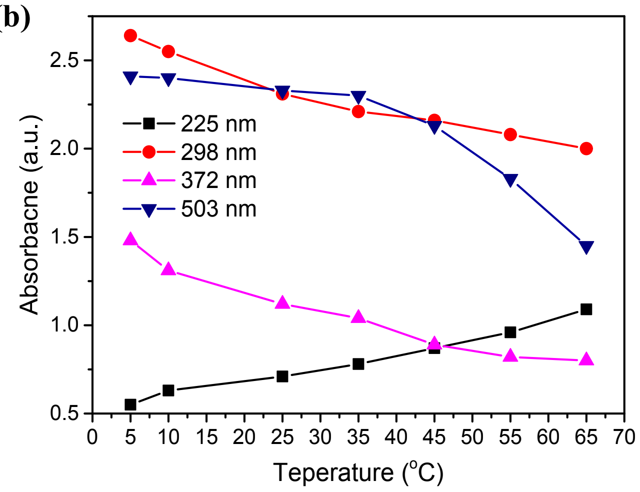

Figure S9. (a) UV-vis spectra of KI (1.4 mM), I_2 (2.1 mM) and Me_{18}-α-CD (2.1 mM) at various temperature. Peak at ca. 503 nm was attributed to Me_{18}-α-CD–I_5^-. (b) The peak intensities were plotted with various temperature. Peak intensities at 370 and 503 nm decreases with increasing temperature. This result shows that the ΔH of host–guest interaction of Me_{18}-α-CD and pentaiodide is negative. This result is in good agreement with previous literature.

2.5. Raman spectroscopy

Raman spectra were recorded on a micro-Raman spectrometer (Jasco NRS 3100KK) equipped with YAG laser (power 1.5 mW, Wavelength 532 nm) and a thermoelectrically cooled CCD detector (DU401-BV-120, Andor). All the measurements were performed at ambient temperature.

UV-vis spectra reveal that I_3^-/I^- aqueous solution in 3 mM α-CD and 2.1 mM Me_{18}-α-CD possess the maximum I_3^- and I_5^- respectively, thus the Raman spectra were recorded at such conditions and the corresponding fittings are shown in Fig. S10. A triiodide cation shows a strong peak at ca. 110 cm^{-1} and a weak peak at ca. 140cm^{-1},[6–10], which are attributed to the symmetric (ν_1) and asymmetric (ν_2) stretch vibration respectively. Another weak peak at 160 cm^{-1} is assigned to the Fermi resonance between ν_1 and the overtone of bending mode (ν_3) of triiodide, though the ν_3 is proposed to be at 50-70 cm^{-1} while it is very weak and not observed in the spectra.[7,9,11] When 3 mM α-CD was added to the I_3^-/I^- solution, the increment of the peak intensity of ν_1 indicates that the host–guest interaction. A new peak appeared at ca. 170 cm^{-1}, which was assigned to the I–I stretch mode and a fraction of the captured I_3^- in α-CD should be described as an adduct of $I^-{\cdot}I_2$ ($\nu_{I\text{-}I}$ = 170 cm^{-1}).[7,9,11] In the case of Me_{18}-α-CD as a host, the intensity of ν_1 peak decreased and the of a peak at 170 cm^{-1} significantly enhanced. I_5^- in solid state exists as an adduct of $I^-{\cdot}2I_2$ or $I_3^-{\cdot}I_2$,[7–9,12,13] and a peak at 170 cm^{-1} was enhanced in the case of $I^-{\cdot}2I_2$. Thus, Me_{18}-α-CD stabilizes the $I^-{\cdot}2I_2$ adduct in aqueous solution.

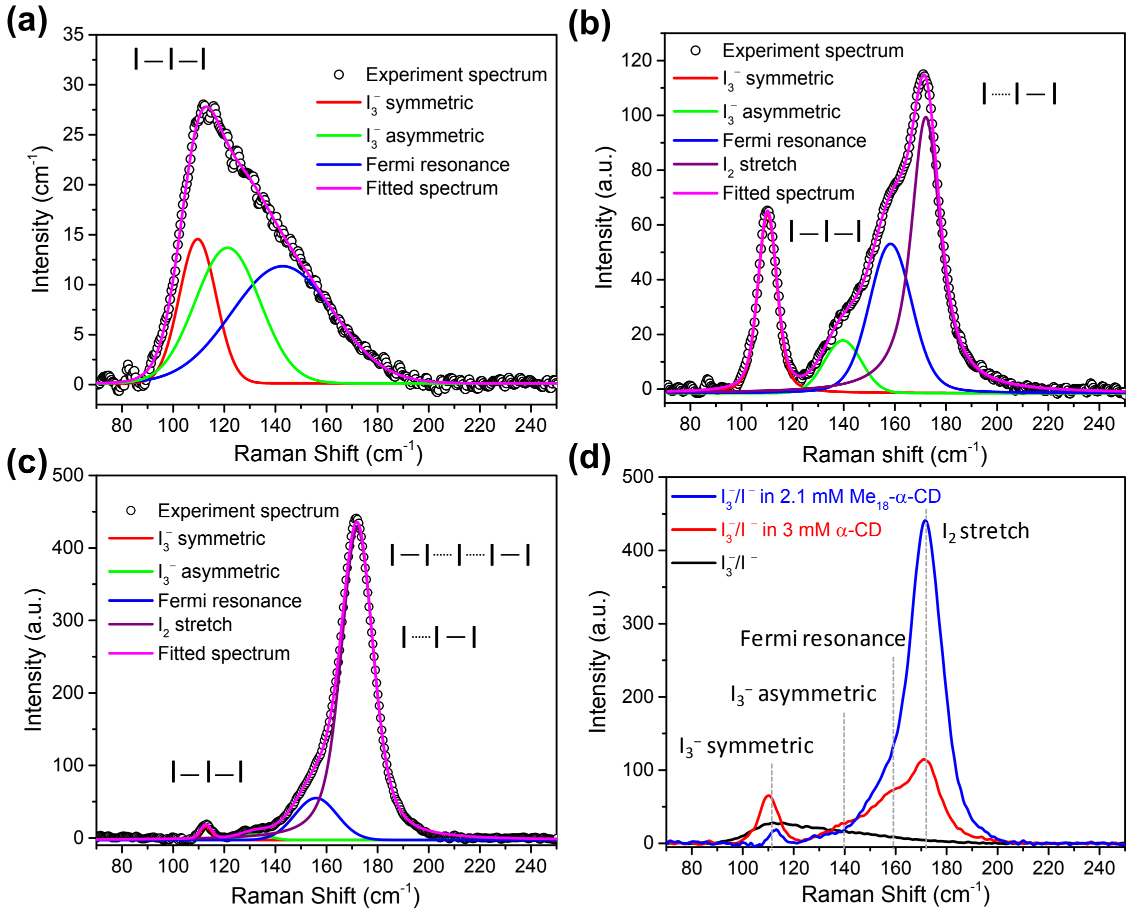

Figure S10. Raman spectra of (a) aqueous solution of KI (12.5 mM) and I2 (2.5 mM), (b) 3 mM α-CD in addition of (a) and (c) 2.1 mM Me_{18}-α-CD in (a). Each spectrum was fitted with Pseudo Voigt functions. All experimental data was overlapped in (d).

2.6. Power output measurement of the KI/I2/M18-α-CD thermocell

(a)

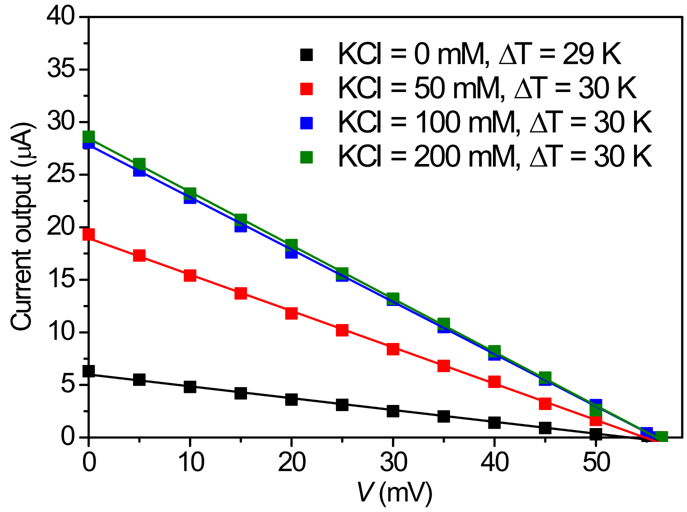

(b)

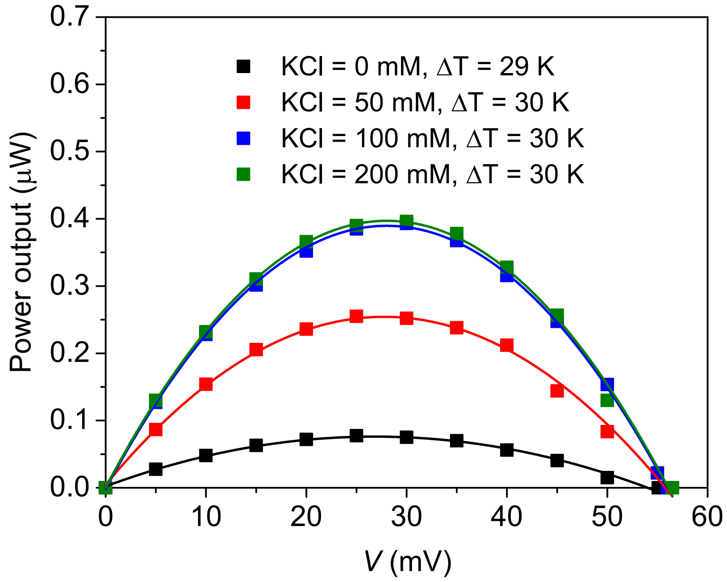

Figure S11. (a) Current output and (b) power out of the Me_{18}-α−CD-based supramolecular thermocell. Temperature difference (ΔT) was controlled between 29−30 K, and the supporting electrolyte KCl was increased from 0 to 200 mM. Errors in the figure are smaller than the size of the squares.

References

1 Z. Liu, A. Samanta, J. Lei, J. Sun, Y. Wang and J. F. Stoddart, *J. Am. Chem. Soc.*, 2016, **138**, 11643–11653.

2 F. Harata, K., Uekama, K., Otagiri, M., Hirayama, *Bull. Chem. Soc. Jpn.*, 1987, **60**, 497.

3 W. Kim, Y. Yamasaki and K. Kataoka, *J. Phys. Chem. B*, 2006, **110**, 10919–10925.

4 E. Freire, L. Obdulio and M. Straume, *Anal. Chem.*, 1990, **62**, 950A–959A.

5

6 F. Bigoli, P. Deplano, A. Ienco, C. Mealli, M. L. Mercuri, M. A. Pellinghelli, G. Pintus, G. Saba and E. F. Trogu, *Inorg Chem*, 1999, **38**, 4626–4636.

7 P. Deplano, F. A. Devillanova, J. R. Ferraro, M. L. Mercuri, V. Lippolis and E. F. Trogu, *Appl. Spectrosc.*, 1994, **48**, 1236–1241.

8 P. H. Svensson and L. Kloo, *J. Chem. Soc. Dalt. Trans.*, 2000, **951**, 2449–2455.

9 L. A. Bengtsson, H. Stegemann, B. Holmberg and H. Füllbier, *Mol. Phys.*, 1991, **73**, 283–296.

10 E. M. Nour, L. H. Chen and J. Laane, *J. Phys. Chem.* 1986, **77843**, 2841–2846.

11 J. S. Zambounis, E. I. Kamitsos, A. P. Patsis and G. C. Papavassiliou, *J. Raman Spectrosc.*, 1992, **23**, 81–85.

12 P. Deplano, J. R. Ferraro, M. L. Mercuri and E. F. Trogu, *Coord. Chem. Rev.*, 1999, **188**, 71–95.

13 P. H. Svensson and L. Kloo, *Chem. Rev.*, 2003, **103,** 1649-1684.
